# Challenges, Opportunities and Future Directions of Membrane Technology for Natural Gas Purification: A Critical Review

**DOI:** 10.3390/membranes12070646

**Published:** 2022-06-23

**Authors:** Aniqa Imtiaz, Mohd Hafiz Dzarfan Othman, Asim Jilani, Imran Ullah Khan, Roziana Kamaludin, Javed Iqbal, Abdullah G. Al-Sehemi

**Affiliations:** 1 Advanced Membrane Technology Research Centre, Universiti Teknologi Malaysia, Johor Bahru 81310 UTM, Johor, Malaysia; aniqaimtiaz@hotmail.com (A.I.); roziana.kamaludin7@gmail.com (R.K.); 2 School of Chemical and Energy Engineering, Faculty of Engineering, Universiti Teknologi Malaysia, Johor Bahru 81310 UTM, Johor, Malaysia; 3 Centre of Nanotechnology, King Abdul-Aziz University, Jeddah 21589, Saudi Arabia; iqbaljavedch@gmail.com; 4 Department of Chemical and Energy Engineering, Pak-Austria Fachhochshule, Institute of Applied Sciences & Technology, Khanpur Road, Mang, Haripur 22650, Pakistan; imran.khan@fcm3.paf-iast.edu.pk; 5 Research Center for Advanced Materials Science (RCAMS), King Khalid University, P.O. Box 9004, Abha 61413, Saudi Arabia; agmasq@gmail.com; 6 Department of Chemistry, College of Science, King Khalid University, P.O. Box 9004, Abha 61413, Saudi Arabia

**Keywords:** membrane technology, natural gas separation, hollow fiber membrane, braid reinforced membrane

## Abstract

Natural gas is an important and fast-growing energy resource in the world and its purification is important in order to reduce environmental hazards and to meet the required quality standards set down by notable pipeline transmission, as well as distribution companies. Therefore, membrane technology has received great attention as it is considered an attractive option for the purification of natural gas in order to remove impurities such as carbon dioxide (CO_2_) and hydrogen sulphide (H_2_S) to meet the usage and transportation requirements. It is also recognized as an appealing alternative to other natural gas purification technologies such as adsorption and cryogenic processes due to its low cost, low energy requirement, easy membrane fabrication process and less requirement for supervision. During the past few decades, membrane-based gas separation technology employing hollow fibers (HF) has emerged as a leading technology and underwent rapid growth. Moreover, hollow fiber (HF) membranes have many advantages including high specific surface area, fewer requirements for maintenance and pre-treatment. However, applications of hollow fiber membranes are sometimes restricted by problems related to their low tensile strength as they are likely to get damaged in high-pressure applications. In this context, braid reinforced hollow fiber membranes offer a solution to this problem and can enhance the mechanical strength and lifespan of hollow fiber membranes. The present review includes a discussion about different materials used to fabricate gas separation membranes such as inorganic, organic and mixed matrix membranes (MMM). This review also includes a discussion about braid reinforced hollow fiber (BRHF) membranes and their ability to be used in natural gas purification as they can tackle high feed pressure and aggressive feeds without getting damaged or broken. A BRHF membrane possesses high tensile strength as compared to a self-supported membrane and if there is good interfacial bonding between the braid and the separation layer, high tensile strength, i.e., upto 170Mpa can be achieved, and due to these factors, it is expected that BRHF membranes could give promising results when used for the purification of natural gas.

## 1. Introduction

Natural gas is formed deep beneath the earth’s surface and is a source of fossil energy. It consists of various compounds (Table 1). Methane (CH_4_) is the largest component of natural gas consisting of one carbon and four hydrogen atoms. It also contains small amounts of natural gas liquids which are called hydrocarbon gas liquids, as well as non-hydrocarbon gases such as water vapor and carbon dioxide (CO_2_). Natural gas is a widely used energy source that is used for heating, cooking, and generation of electricity, as well as fuel for vehicles. The purification of natural gas is important due to environmental hazards. Natural gas conditioning involves the elimination of acid gases such as CO_2_ and hydrogen sulphide (H_2_S), besides water vapor. The requirement for environment-friendly and energy-efficient gas purification techniques has encouraged extensive research into membrane-based gas purification technology. This technology is widely used in the separation of CO_2_ from natural gas. The removal of CO_2_ among other impurities is important because of its corrosive nature and its uncontrolled emission into the atmosphere has become a serious concern as it is hazardous for human health and also leads to climate change and flooding.

Many different technologies were available on an industrial scale for natural gas purification to ensure the removal of CO_2_. These technologies include adsorption, absorption, and membrane separation with their own advantages and disadvantages. Among available separation methods, membrane separation technology has come out to be a feasible option over other technologies because of advantages such as economy [3,4], process safety, and low energy requirement, as well as less requirement for supervision [5]. It was observed that most of the polymeric membranes experience a trade-off between selectivity and permeability as polymers that are more permeable are less selective and this, in turn, results in Robeson Upper bound. Inorganic fillers such as zeolites [6], metal-organic frameworks (MOFs) [7], carbon nanotubes [8], mesoporous silica, and carbon molecular sieves [9] were introduced into the polymer matrix to overcome this hurdle and to fabricate mixed matrix membranes (MMM). The selection of inorganic filler in order to fabricate MMM is quite challenging owing to their textural characteristics as well as their interaction with polymers [10]. The incorporation of MOF into the polymeric matrix for MMM fabrication can provide a major opportunity in solving trade-off complication that is normally faced by polymeric membranes. They have been considered an attractive filler for MMM fabrication due to their 3D coordination network with porosity, as well as their good compatibility with the polymeric matrix [11]. They exhibit better compatibility with the polymer matrix as organic linkers that are present in MOF have strong interaction with polymer chains.

During the past few decades, membrane separation by employing hollow fiber (HF) membrane has become one of the most evolving technologies and experienced rapid growth. Hollow fiber membranes have managed to gain commercial interest with numerous applications at the forefront of experimental research in order to carry out purification of drinking water, bioseparations, and treatment of wastewater besides liquid phase and gaseous separations. Hollow fiber membranes are also playing a major role in gas separation applications due to them having high selectivity and separation areas. They are considered attractive for industrial use as they are self-supporting and can easily be assembled into modules [12,13]. Hollow fiber membranes that are fabricated by the immersion-precipitation technique have higher permeability but they suffer from low mechanical strength because of a loose support layer and dense layer [14]. Hence, HF membranes are likely to get damaged by high pressure or high air flow. For its use in high-pressure requiring applications, it is important to enhance the mechanical strength of HF membranes. For this purpose, the coating of a separation layer onto a higher strength tubular braid is considered to be a productive approach [15]. The production of reinforced fiber membranes has not been adequately investigated in the literature. The concept of braid reinforced hollow fiber (BRHF) membranes was first explained by Cooper et al. [16] who used a casting bob to make reinforced fibers and described the usage of embedded braided material. However, this technique turned out to be impractical for fabricating capillary membranes. The concept of a semi-permeable composite membrane was described by Hayano et al. [17], which consisted of a porous substance as well as supporting material made up of fibrous material that is embedded in a porous substance wall. The concept of current technology was introduced by Lee et al. [18] who fabricated a braid reinforced HF membrane in which a thin film of polymer resin was coated on the surface of the reinforcing tubular braid. Because of the superb mechanical strength of threads or fabric, they can also be used as an alternative to tubular braid as a supporting or reinforcing material for hollow fiber membranes [19].

This review highlights the ways that could enhance the separation performance of membranes in natural gas purification. Mixed matrix membranes that comprise inorganic filler dispersed in a polymer matrix and combine the advantages of both polymeric and inorganic membranes offer an interesting approach to improving the separation performance. This review also highlights the fabrication of braid-supported HF membranes along with their applications. Moreover, it discusses the significance of polymer, braid and spinneret types employed for the fabrication of BRHF membranes and their effects on morphology as well as the performance of fabricated BRHF membranes.

## 2. General Processes of Gas Purification

Natural raw gas consists of methane (CH_4_) with other light gases such as butane (C_4_H_10_), propane (C_3_H_8_), ethane (C_2_H_6_) and corrosive gases such as hydrogen sulphide (H_2_S) and carbon dioxide (CO_2_) [1]. The typical raw gas composition is given in Table 1.

Traditional techniques consisting of reactive absorption, solid bed absorption and physical absorption are hired in lots of plants throughout the sector for the removal of corrosive gases [20]. Many advantages are gained by using these techniques, but problems associated with operational costs and high capital are also being faced. In the purification of natural gas, the most important and crucial step is the extraction of CO_2_. The high content of CO_2_ present in natural gas streams becomes very corrosive in the presence of water and damages the pipelines and system; hence, it has to be reduced to less than 2%. Thus, the technologies for the separation of CO_2_ have attracted the interest of researchers worldwide [21]. It is essential to have the selection of appropriate technology by considering the economy and efficiency for a specific application. In order to obtain almost pure CH_4_, the natural raw gas is refined in different stages. Absorption, adsorption, cryogenic separation and membrane technology are the currently developed technologies available for natural gas purification at an industrial level [20]. These technologies are used for the separation of CO_2_, while for the reduction in high concentrations of contaminants such as H_2_S, there is a need for a pre-upgrade stage.

**Figure 1:**
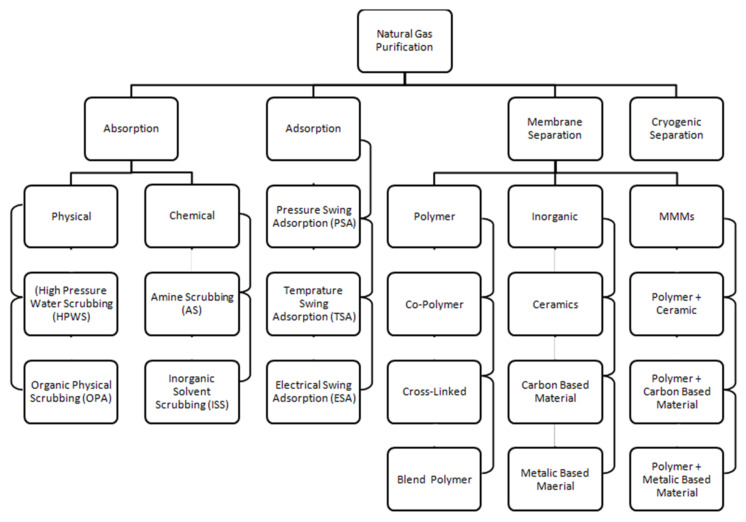
Natural gas purification technologies [23]. Reprinted/adapted with permission from Ref. [23]. Copyright 2022, Elsevier.

Classification of the technologies used to purify natural raw gas is shown in Figure 1 below. Among those processes, the membrane separation process is the best in energy efficiency and offers the least processing cost [22].

### 2.1. Absorption

Absorption relies upon the solubility of different components of gas in a liquid solvent. Liquid solvent’s counter flow meets raw gas in a column which is filled up with packing material in order to increase the area of contact between liquid and gas. Carbon dioxide has a greater solubility in liquid than CH_4_; therefore, the gas that exits from the column has a greater CH_4_ concentration and liquid having a high concentration of CO_2_ leaves the column [24]. Physical absorption is divided into two types, i.e., organic physical scrubbing and also high-pressure water scrubbing. While the types of chemical absorption include inorganic solvent scrubbing and amine scrubbing [25].

#### 2.1.1. High-Pressure Water Scrubbing

To remove H_2_S and CO_2_ from raw gas high-pressure water scrubbing is used, which is one of the most well-established and common technologies as these gases are readily soluble in water compared to CH_4_. The operating pressure for water scrubbing is 10 bar, and the gas enters from the bottom of the column and water is then introduced counter-currently [26]. Henry’s law governed the physical absorption of gases, which reveals that the amount of gas that is dissolved in a specified volume and type of liquid at constant temperature is proportional to its partial pressure in equilibrium with the liquid. Moreover, it is determined that carbon dioxide solubility increases at low temperatures [27]. This process is also helpful to remove H_2_S as it is more soluble in water than CO_2_ [28].

**Figure 2:**
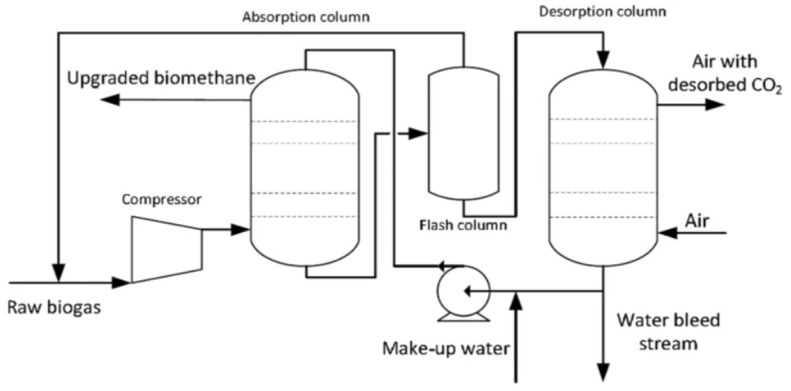
Process flow diagram of water scrubber [31]. Reprinted/adapted with permission from Ref. [31]. Copyright 2022, John Wiley and sons.

The raw gas is fed into the bottom of the absorption column as shown in Figure 2 at an operating pressure of 10 bar and temperature of 35–40 °C and in order to increase the gas–liquid area of contact, it is normally filled with random packing and water is introduced at the top of the column. Counter current flow of liquid and gas is necessary to assure high efficiency. CO_2_ and a very little amount of CH_4_ are absorbed in water. The process selectivity is highly dependent on the high solubility of CO_2_ in water as compared to CH_4_. In a flash column, to reduce the loss of methane to the off-gas stream from the water scrubber, the pressure is reduced to 3 bar. On exiting from the scrubber’s bottom, wastewater is fully saturated with H_2_S and CO_2_ and a little quantity of CH_4_ which is restored and reversed back to the absorption column. By decreasing pressure and by air stripping in the desorption column, regeneration is achieved. If the concentration of H_2_S is high, air stripping is not suggested because the water will quickly get contaminated with sulphur which in turn causes operational issues along with corrosion. The use of fresh water is recommended if there is an availability of the cheap source of water. In the water scrubbing process, pre-removal of H_2_S is mandatory when there is a high concentration of H_2_S [29]. Although it is an efficient and eco-friendly process having a high recovery of methane (>97%) and no requirement of special chemicals, higher operational cost and high investment are required. In addition, during the water regeneration process, high consumption of energy is required which leads to excessive costs [30].

#### 2.1.2. Chemical or Amine Scrubbing Process

A reversible reaction among solvents and absorbed substances are involved in chemical absorption. Methyl diethanolamine (MDEA), monoethanolamine (MEA) and diethanolamine (DEA) are some widely recognized amines utilized as solvents for eliminating acidic gases (CO_2_ and H_2_S). In this process, a blend of piperazine (PZ) and MDEA also known as activated MDEA (AMDEA) is mostly used [32]. The absorption capacity of MDEA is significantly lesser than AMDEA. The reason is the presence of the tertiary amine in MDEA and primary and secondary amines presence in PZ which provides a relatively higher reaction rate for absorption of CO_2_.

**Figure 3:**
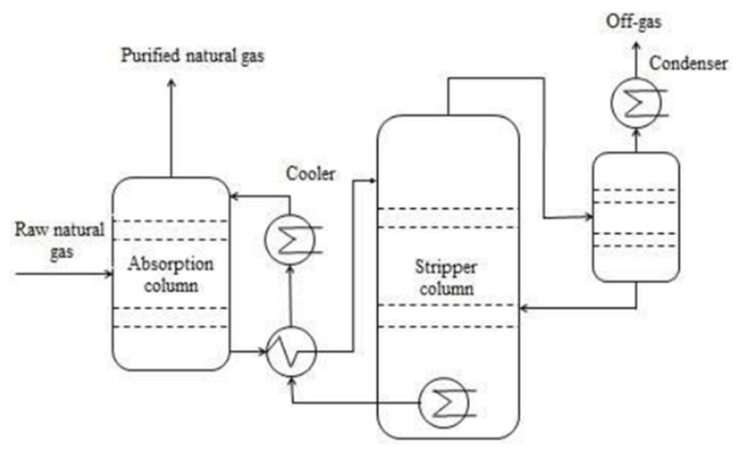
Process flow diagram of amine scrubber [31]. Reprinted/adapted with permission from Ref. [31]. Copyright 2022, John Wiley and sons.

Mostly, the system of amine scrubber comprises an absorber as shown in Figure 3, in which from natural gas, CO_2_ is absorbed and it also has a stripper in which by heating under reduced pressure, separation of CO_2_ from the waste amine solution is carried out [33]. Within the absorber, natural gas enters from the bottom and to make a counter-current flow contact, an amine solution is introduced from the column’s top. Amine solution and the CO_2_ in the gas react with each other and get absorbed. This reaction is exothermic in which the absorber’s temperature increases from 20–40 to 45–65 °C [34]. Normally, with decreasing temperature the CO_2_ solubility increases in H_2_O [27] but in the case of amine scrubbing (AS), an increase in temperature increases the reaction rate among the amine solution and the CO_2_, and therefore gives increased absorption of CO_2_. CH_4_ exits from the column’s head and 1–2 bar is the absorber’s operating pressure [31]. The liquid from the absorber’s bottom goes through the heat exchanger and is pumped to the top of the stripper, where CO_2_ is released after contact with steam. Amine solution is boiled in a reboiler at 120–150 °C present at the lower part of the stripper column [31]. Reboiler gives the heat of reaction for recovery of amine solution and for CO_2_ release from waste amine solution. Very concentrated CH_4_ gas of >99% purity is achieved, accompanied by less operational and higher investment cost, and most importantly there is a need for massive heat to regenerate the amine solution [35]. Amine solution absorbs H_2_ S that is present in the raw gas but for regeneration, a high temperature is needed in order to desorb H_2_S. So, it is more suitable to remove it before the process of amine scrubbing. The necessity to treat waste chemicals, corrosion and building up of contaminants is another disadvantage of this process which makes the process of amine scrubbing more complex [36].

#### 2.1.3. Organic Physical Scrubbing (OPS)

**Figure 4:**
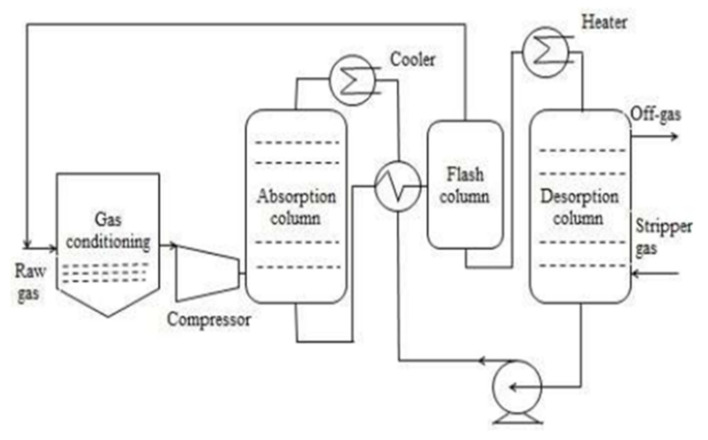
Flow diagram of organic solvent scrubber [31]. Reprinted/adapted with permission from Ref. [31]. Copyright 2022, John Wiley and sons.

This process is quite identical to water scrubbing, but instead of water, an organic solvent is used in this process. Polyethylene glycol ethers (PEG), *N*-methyl-2-pyrrolidone (NMP), and methanol (CH_3_OH) are some different organic solvents used for the absorption of CO_2_. Genosorb^®^ and Selexol^®^ are the brand names of PEG liquids employed in organic physical scrubbing [35]. In the same upgrading capacity, the solubility of CO_2_ in PEG is five times higher than in water [37], which results in less pumping requirement and lower organic solvent demand [38]. As compared to the water scrubber, the volume of solvent that is to be recirculated back to the system decreases due to the increase in solubility of CO_2_ in the solvent. An organic physical scrubber is shown in Figure 4.

Before getting injected into the bottom of the absorption column the raw gas is cooled and compressed to 6–8 bars. To generate counter-current liquid and gas flow, the organic solvent is introduced from the column’s top. In order to maintain a low temperature of 20 °C in the absorption column, the organic solvent is also cooled before getting injected into the column. The organic solvent leaving the lower part of the absorption column is heat exchanged with the organic solvent which will be injected into the column’s top. The organic solvent is then injected into the flash column, where under reduced pressure some amount of CO_2_ and dissolved CH_4_ is discharged and recirculated towards the inlet of raw gas. For organic solvent regeneration, before entering the column of desorption it is heated further to about 40 °C. The solvent is injected into the column’s top and pressure is lowered to 1 bar. The solvent that is regenerated is then introduced to the top of the absorption column. The heat that is required in this process is waste heat and can be produced by regenerative thermal oxidation (RTO) unit and compressor which oxidizes CH_4_ in off-gas. Despite the fact that in terms of CO_2_ removal, this process is more efficient than water scrubbing but for the regeneration of solvent more energy is required. Moreover, the expense is higher for organic solvents than that for water [37].

### 2.2. Cryogenic Separation

**Figure 5:**
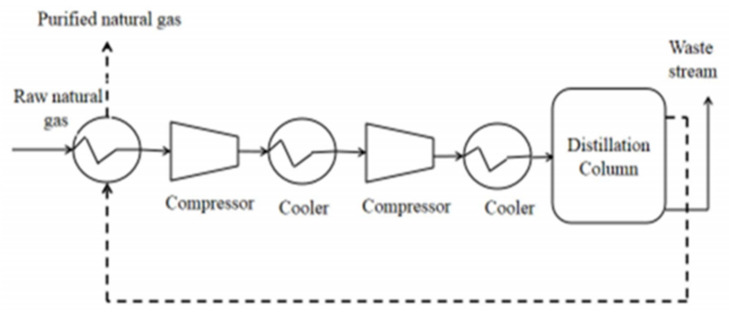
Process flow diagram of cryogenic separation process [23]. Reprinted/adapted with permission from Ref. [23]. Copyright 2022, Elsevier.

The principle on which this separation is based is that different gas such as H_2_S and CO_2_ liquefies under different conditions of pressure and temperature. It works under the conditions of high pressure (80 bar) and low temperature (−170 °C). The boiling point of CO_2_ is −78.2 °C which is much higher than the boiling point of CH_4_, and liquefying CH_4_ allows CO_2_ to separate from CH_4_ [39,40]. A series of heat exchangers and compressors are used to maintain the operating conditions as shown in Figure 5. The usage of different equipment, mainly distillation columns, heat exchangers, compressors and turbines is the primary disadvantage of this process because it causes an increase in operational costs and capital with high requirements of energy [41]. For the purification of raw gas, four stages are involved in a common cryogenic system. In the first stage, halogens, dust particles, moisture, H_2_S, siloxanes and other components that are not necessary are removed. The gas in the second step is compressed to 1000 kPa and cooled to −25 °C subsequently. The gas is further cooled until −55 °C in the third stage of this process and CO_2_ which is in the liquefied form is eliminated from the mixture of gas. The remaining stream of gas is then cooled further until −85 °C is achieved and CO_2_ reaches solid form which is then finally removed in the final and last stage. The refined gas is then depressurized and can be utilized in different applications. This separation system is viewed as an under-developed technique but some commercial plants are operational [42]. If the goal is to produce liquefied natural gas (LNG) and liquefied biomethane (LBM), cryogenic separation can be beneficial [35]. Pre-separation of H_2_S and H_2_O is recommended to avoid equipment plugging caused because of water freezing in raw gas [29]. The operating pressure of the system is reduced by this phenomenon [43].

### 2.3. Pressure Swing Adsorption

Pressure swing adsorption is a dry technique used to separate gases through contact between the adsorbent and the gas molecules. The adsorbents that are used have high specific areas to maximize the contact between gas and adsorbent and are porous solids. The adsorbents used are usually of kinetic type, adsorbing CO_2_ at a faster rate than CH_4_ because of controlled diffusion rates or equilibrium type, adsorbing a huge load of CO_2_ than CH_4_. Commonly used materials include activated carbons, titanosilicates, carbon molecular sieves (CMS), natural and synthetic zeolites as well as silica gels. H_2_S must be removed from raw gas before introducing it to adsorption columns as it will bring irreversible damage to the adsorbents [44]. The pressure swing adsorption system typically consists of four phases, i.e., pressurization, feed, blowdown and purge. The raw gas is pressurized at 5–10 bar and fed to the column during the feed phase. In the column bed, CO_2_ is adsorbed while CH_4_ that is unaffected by adsorbent passes through the column. The inlet is closed when the column bed is full of CO_2_, and the blowdown phase begins. In order to desorb CO_2_ from the adsorbent, the pressure is reduced, and CO_2_-rich gas is expelled from the column. Along with the desorbed CO_2_, some methane is also lost. Finally, the purge phase begins at the lowest column pressure. In order to empty the column from the CO_2_ that is desorbed from the bed of the column, upgraded gas is blown through it. With an upgraded or raw gas, the column is regenerated and then repressurized [45].

**Figure 6:**
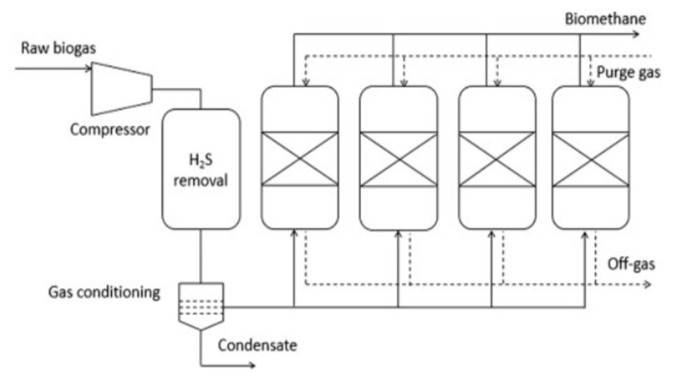
Process diagram for PSA system [23]. Reprinted/adapted with permission from Ref. [23]. Copyright 2022, Elsevier.

A PSA system usually consists of two to four columns as shown in Figure 6 and among them, one of the columns is involved in adsorption, while the other columns are involved in regeneration. These columns are interconnected to minimize the methane loss and the during the blowdown phase, the gas flow from one column is utilized to pressurize another column in the pressure equalization phase. This phenomenon also reduces the consumption of energy during the process.

### 2.4. Membrane Separation

Before the 1800s when synthetic nitrocellulose membrane was not prepared, biological membranes were made up of pig’s bladder, cattle, plant (onion skin) and fish [46]. In 1831, it was identified by a pioneer researcher named Mitchel that various gases permeate at different rates through natural rubber film [47]. In 1855, Fick’s law of diffusion was presented by Adolf Fick and he also made synthetic membranes using cellulose nitrate [48]. Furthermore, the permeation of gas through membranes was studied by Thomas Graham, and he then proposed Graham’s law of diffusion through micro porous membranes [49]. However, still, studies were restricted to theories, experimental stages and laws without commercial applications due to low fluxes of gas. A major development in membrane-based separation technology was made by Loeb and Sourirajan [50]. A skin-type polymeric membrane with a porous sublayer from cellulose acetate was prepared by them in one single step with the process of “phase inversion”. Later, to prepare other polymeric membranes, this process was used frequently. High-flux asymmetric membranes with various modules such as hollow fibers and spiral wounds were produced by this procedure [51]. However, because of the presence of tiny defects and pinholes on the outer surface, these membranes suffered low fluxes [5]. An innovative solution was presented by Henis and Tripodi [42] by applying a thin composite coating layer of highly permeable elastomer on the surface of the membrane to overcome these deficiencies and defects [52]. By this method, a thin and defect-free selective layer was produced with a high separation performance. To use the membrane commercially for the process of industrial gas separation, a great potential was generated by this approach. The process of membrane-based gas separation has become a part of the market share during the last 40 years [53,54]. Membrane-based gas separation technology has successfully gained considerable attention from different sectors mainly academics and industries in their exploration and research as it aids in reducing costs and environmental issues. Membrane behaves as a permeable barrier that permits some components to pass and also controls their permeability that is primarily dependent on the driving forces applied such as difference in pressure, concentration, electric charges and temperature of several species. In order to explain the process of membrane separation two models pore-flow model and the solution-diffusion model are generally used [55]. In the solution-diffusion model, due to the difference in concentration, permeates are dissolved in the material of the membrane and then diffuse across the membrane. Later in this process, the separation of permeates is carried out via pressure-driven convective flow by small pores [56]. However, for the transportation of gas in polymeric membranes, the solution-diffusion model is used frequently [57,58]. A membrane’s commercial value is normally determined by its transport properties, i.e., selectivity and permeability [59]. The membrane’s selectivity to specific liquid or gas molecules is dependent upon the ability of molecules to diffuse across the membrane. The ideal separation factor or permselectivity is defined as the ratio of pure gas permeability of gases that are being separated. Ideally, membranes that are to be employed in separations should have higher permeability as well as selectivity. If the permeability of the membrane is higher, a lesser membrane area is needed for a given separation and hence the membrane cost will be lower. Furthermore, if the selectivity of membrane is higher, methane losses would be lower, and hence, high volume of product can be recovered [59,60].

## 3. Membranes for Gas Purification

Because of the extensive use of natural gas, the purification of natural gas has become a broad and wide-reaching gas separation process. Although the traditional amine absorption process undoubtedly can remove a high percentage of impurities, it still suffers from different drawbacks such as higher capital costs, higher energy consumption, complex operation and corrosion of equipment, etc. [61,62]. Likewise, the processes that are developed for the separation of N_2_ such as pressure swing adsorption and cryogenic separation also suffer from problems related to energy consumption. Hence, the rapid and successful development of membrane-based gas separation technology has provided us with a convenient, energy-saving and economical separation process [63].

**Figure 7:**
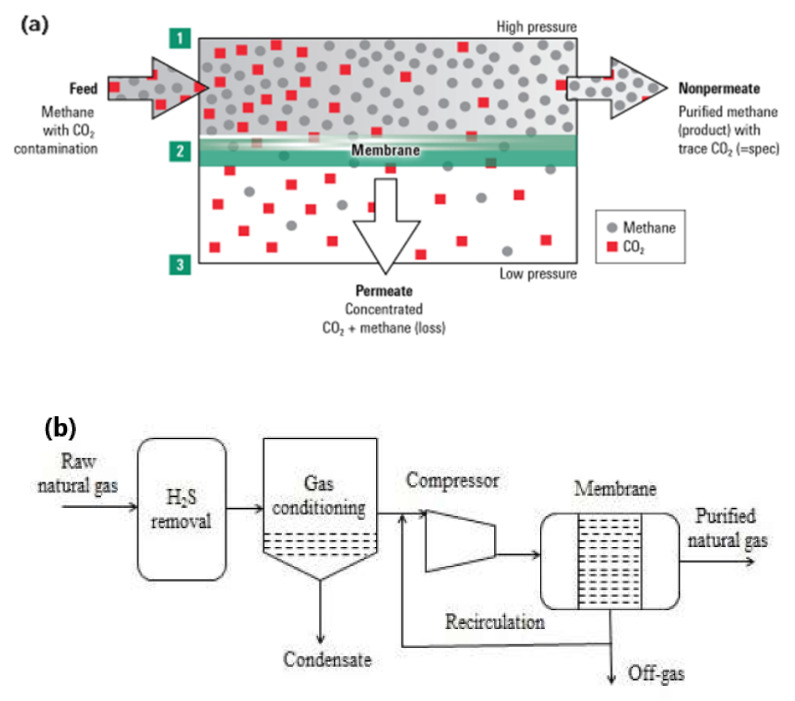
(**a**) Illustration of membrane-based gas separation process [23]. (**b**) Flow diagram of membrane separation process [23]. Reprinted/adapted with permission from Ref. [23]. Copyright 2022, Elsevier.

In the case of purification of natural gas, CO_2_ can permeate through the membrane and at the same time CH_4_ gets retained at the feed side as retentate as shown in Figure 7a. Membrane-based gas separation can be more advantageous if gas flow is lower and CO_2_ content at the inlet is high. These factors are quite favorable for standard natural gas purification units. The membrane-based gas separation technology is mostly used commercially because of its advantages such as better selectivity, low energy consumption as well as easily engineered modules. With this technology, high CO_2_ purity can be achieved along with efficient CH_4_ recovery up to 96%. This gas separation technology is based on gas dissolution and diffusion into polymeric membranes. Gas transportation across the film occurs by applying differential pressure on opposite sides of the polymer film. The rate of gas permeation is mostly controlled by the diffusion coefficient and solubility coefficient of the membrane system. Three types of membranes that are normally used for the gas separation process include polymeric, inorganic and mixed matrix membranes.

### 3.1. Membrane Materials

Normally in the field of membrane-based gas separations, attention is focused particularly on the permeability (productivity), as well as the selectivity (efficiency) of the membrane for a specified gas separation process [64]. Hence, in order to attain high permeability and selectivity, several types of membrane materials have been investigated and are classified into three types namely polymeric, inorganic and mixed matrix membranes [65]. An appropriate choice of membrane materials does not merely contribute to attainting higher permeability ratios, but also aids in yielding superior permeabilities. A particular membrane chemistry is also of great importance and is dependent upon the type of separation that is to be achieved. Hence, the selection of appropriate membrane material is an area of great importance. Moreover, it is worth mentioning that asymmetric membrane configuration facilitates membrane applicable for industrial applications.

#### 3.1.1. Polymeric Membranes

Most of the membranes that are used commercially are polymeric and are made of organic materials such as cellulose acetate (CA), polysulfone (PSF), polydimethylsiloxane (PDMS), polycarbonate (PC) and polyimide (PI) [66,67]. These membranes are easy to fabricate, have high selective permeation and have great mechanical strength. A total of 98% CH_4_ purity was achieved by testing polyvinyl amine/polyvinyl alcohol blend membrane [53]. For the purification of gas, the first commercialized polymeric membrane was the CA membrane which removes CO_2_ and H_2_S [68]. Cellulose acetate is inexpensive because of the renewable and plentiful resources of cellulose having remarkable separation properties. The utilization of CA membranes is restricted in gas separation because it possesses several limitations. The cellulose acetate membrane exhibited a gas mixture selectivity that was lower than the ideal selectivity which is calculated for neat gas due to plasticization phenomena [69]. These membranes are susceptible to plasticization (plasticization = 8 bar) [70] because of the –OH functional group, which helps CO_2_ to get easily dissolved in the membrane matrix. PDMS (polydimethylsiloxane) due to its higher gas permeability in comparison with other synthetic polymers was viewed as a remarkable candidate [71]. Permeability of CH_4_ and CO_2_ in PDMS is relatively greater than others because of the existence of many configurations as well as the composition of the side chain. The obvious disadvantage of this kind of material is the low separation factor as well as low mechanical strength [72].

**Figure 8:**
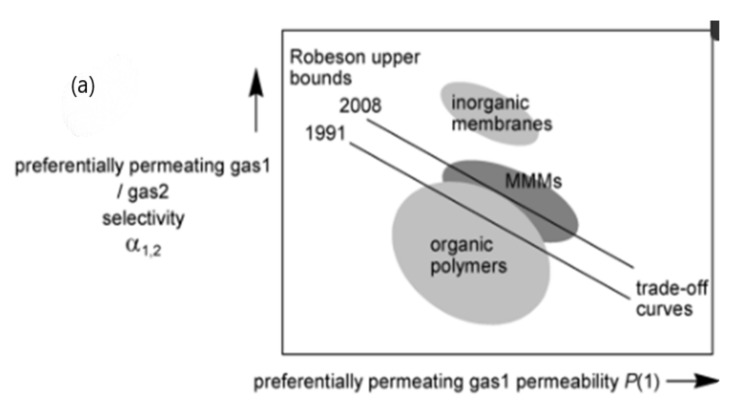

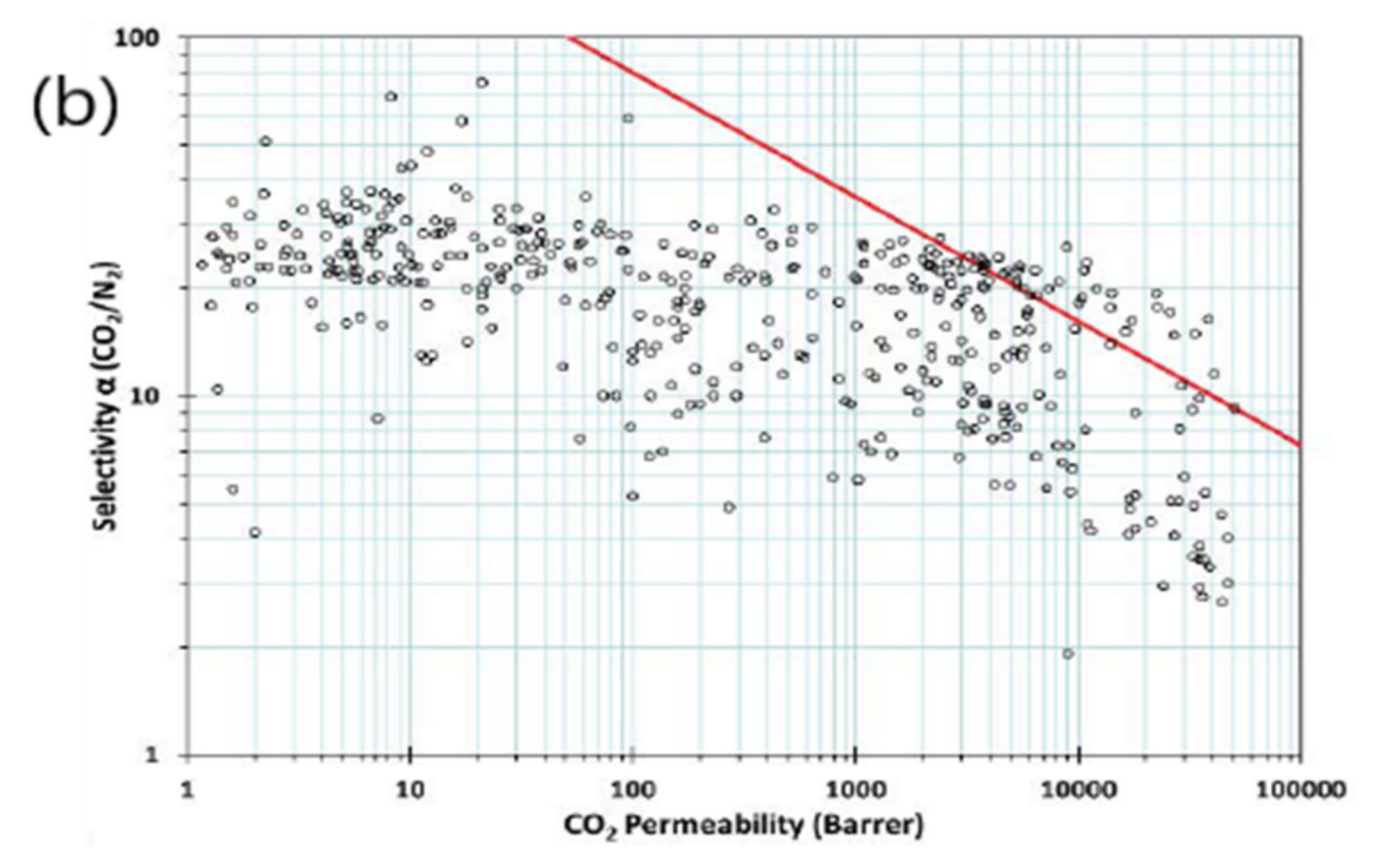
(**a**) Robeson trade-off limit between permeability/selectivity [77]. Reprinted/adapted with permission from Ref. [77]. Copyright 2022, John Wiley and sons (**b**) Robeson plot for gas pair (CO_2_/N_2_). Red line depicts upper bound 2008. Vacant symbols display the polymers that were listed in membrane society of Australasia [73].

In the past, several polymers have been synthesized and tested but unfortunately, only a few of them hit the market. Most of the commercial membranes are polymer-based and have low permeability and high selectivity. Low permeability can be related to the productivity of membrane-based gas separation process, hence, lower permeability makes them unsuitable for the treatment of large amounts of gas, for instance, treatment of flue gases. Though, the fabrication of membranes possessing higher permeability as well as selectivity is a challenging task because of the trade-off problem between permeability and selectivity. The trade-off was first proposed by Robeson [73], also it is evident in well-known log-log plot where gas pair (CO_2_/N_2_) selectivity is outlined as a function of the permeability of more permeable gas (CO_2_) as shown in Figure 8b. For example, a suitable membrane that is to be employed for capturing CO_2_ from a flue gas power plant would essentially require a polymer having a permeability of 1000 barrer at least, and selectivity of (CO_2_/N_2_) over 30 [74]. At this time, only a few of these polymers are somehow close to this mark. Among newly synthesized polymers, thermally rearranged polymers and polymers of intrinsic microporosity are most leading ones [75,76]. In spite of them having higher permeability, their physical aging along with their costly multistep synthesis are their main drawbacks and these drawbacks should be resolved prior to their use in industrial processes.

In the separation of H_2_S and CO_2_ from natural gas, state-of-the-art polymeric membranes are competitive economically as compared to conventional technologies in operating and capital costs [25]. Table 2 below comprises commercial membrane materials along with their selectivities in order to remove impurities from natural gas. As mentioned above, polymeric membranes suffer severe drawbacks, even though promising results are exhibited in gas separation. The major inconvenient loss as mentioned earlier was low membrane selectivity, it demands a separation system that has multi-stages and which imparts higher capital cost. Moreover, performance is not commonly maintained by polymeric membranes, as in the extreme environmental conditions of high pressure and temperature, it deteriorates. The main cause of the problematic phenomena is chain swelling in the existence of components that are extremely corrosive in feed. Other problems include compaction, aging of membranes and plasticization. As shown in Figure 8a, membranes that are extremely permeable are followed by low selectivity of gas pairs. Table 3 below states some of the advantages and disadvantages of polymeric membranes.

#### 3.1.2. Inorganic Membranes

Inorganic membranes offer more thermal stability, are resistant to the chemicals and also offer better mechanical strength, so they are considered more advantageous than conventional polymeric membranes. They are normally made using zeolites, carbon molecular sieves (CMS), metal-organic frameworks and ceramics [81]. As compared to polymeric membranes, inorganic membranes exhibit high selectivity and gas fluxes, e.g., CMS and zeolites have higher selectivity and diffusivity than polymeric membranes. Their excellent selectivity is due to well-defined shape and size discrimination which in turn leads to narrow pore size distribution [82]. Most of the inorganic membranes exceeding the Robeson upper bound facilitate the selectivity and permeability Figure 8a.

Inorganic membranes have many advantages such as solvent resistance at high-pressure conditions as well as stability at high-temperature conditions (Table 4). Inorganic membranes also have some drawbacks such as high fabrication and operational cost, they have low surface area per unit volume, and for industrial use it is difficult to transform them into modules with large surface area [82]. It is observed that inorganic membrane fabrication is a tough process and there is a need for continuous monitoring because of their delicate structure [83]. Despite their excellent properties of gas separation, rigid materialssuch as zeolites and carbon molecular sieves (CMS) face problems in forming a continuous zero-defect membrane that may be used in practical applications [1]. Hence, researchers were motivated to develop new materials for membranes as inorganic and polymeric, both membranes have limitations. For gas separation membranes, in order to overcome the issues related to both polymeric and inorganic membranes, researchers then developed new membrane material named Mixed matrix membrane (explained in Section 4).

In general, H_2_S can negatively affect the performance of the membrane, so its pre-removal is necessary. In Figure 7b the process for the purification of natural gas with membrane technology is shown. Before entering the gas in the membrane unit, it is also necessary to remove the oil droplets, water, and aerosols by a filter [27]. A system needs to be developed that can remove CO_2_ as well as H_2_S from raw gas and can also trace the impurities using different membranes. As compared to the single-stage process, the multi-stage process has fewer operating and investment costs as well as gives high purity of CH_4_ [85]. Xiao et al. (2015) [25] identified that by using the multistage process, the recovery of CH_4_ can be improved from 80 to 99.5% [25].

### 3.2. General Membrane Fabrication Procedures

The selection of the production process of the membrane depends on the desired membrane structure and the choice of polymer. Different methods are used to fabricate the membranes, these methods include; interfacial polymerization, phase inversion, track etching, controlled stretching, melt extrusion and electro-spinning [86]. Currently, because of the scalability and flexibility, phase inversion is the most common method [86]. Apart from the method of phase inversion, especially in the membrane contractor applications, the electrospinning method has gained researchers’ interest.

#### 3.2.1. Phase Inversion Method

This technique allows the synthesis of HF and flat sheet membranes. Phase inversion is generally a de mixing process in which polymer solution is transformed into the solid phase under controlled conditions and in this process polymer solution that is thermodynamically stable immediately separates into polymer-rich and polymer-lean phases [87]. This technique can be performed by following steps. (1) Immersing the polymer solution in a coagulation bath to permit solvent exchange phenomena to occur. (2) Thermally induced phase separation (TIPS) in which the exchange of solvent is brought about by high temperature. (3) Vapor-induced phase separation in which desired polymer is prepared by volatile solvent and is then evaporated.

#### 3.2.2. Electrospinning Method

**Figure 9:**
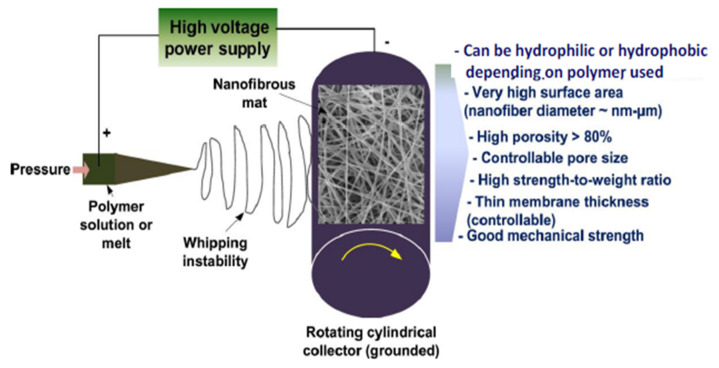
Electrospinning setup [92]. Reprinted/adapted with permission from Ref. [92]. Copyright 2022, Elsevier.

Electrospinning for the production of interconnected and continuous micro/nanofibers from a variety of materials is considered a universal technique [88]. Electrospun nanofiber membranes due to their advantages such as high surface area, high porosity and their controllable pore size have the ability to compete with conventional phase inversion membranes [89,90]. The flexibility involved in the construction of the device used for electrospinning as well as post-treatment process diversity to electrospin membrane allow scientists to modify the structure as well as properties of membranes. Hence, many researchers have paid attention to using this technique for the fabrication of polymeric hollow fiber membranes [91]. The electrospinning technique includes the application of strong electric fields on melt or polymer blend solution that produces nanofibers and deposits them on a grounded collector. The setup used for electrospinning has 3 main constituents as shown in Figure 9. First, is the power supply having a high voltage varying from 0–40 kv [92]. Second, is the container with a needle containing melt or polymer solution. Third, is the grounded collector that may be drum type or flat plate or may have other different configuration designs. The high voltage power supply is provided to the metallic needle after the polymer solution is fed to the syringe. The polymer solution or melt is discharged from the nozzle to the collector as a conically shaped nanofiber when an electric potential greater than the surface tension of the melt or polymer solution is provided. Most solvents are evaporated during their travel from nozzle to collector, leaving behind the dry nanofibers to mound and this results in the formation of electrospun membrane. By changing the processing conditions and shapes of the collector and needle, the membrane’s morphology can be altered as per requirement.

### 3.3. Main Permeation Mechanism

**Figure 10:**
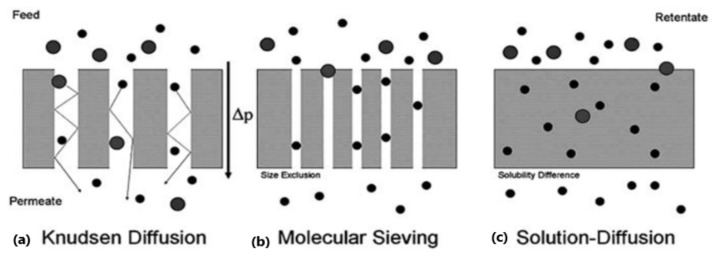
Gas permeation mechanism: (**a**) Knudsen-diffusion mechanism; (**b**) molecular-sieving mechanism; (**c**) solution- diffusion mechanism [95]. Adapted from [95].

**Figure 11:**
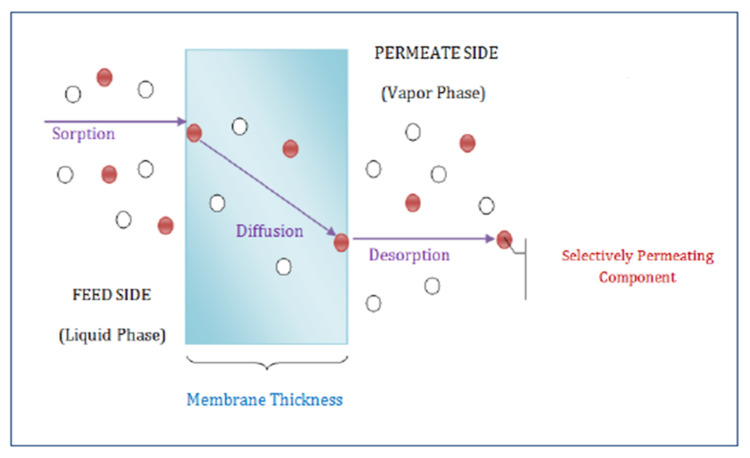
Comprehensive description of solution diffusion mechanism [96]. Adapted from [96].

Separation through a membrane usually takes place according to membrane morphology and is generally based upon different transport/permeation mechanisms. Main mechanisms of transport including Knudsen diffusion, solution diffusion and molecular sieving are proposed for gaseous transport across membranes usually depending upon the membrane matrix porosity [93] as shown in Figure 10 below. (1) Knudsen’s diffusion takes place within a porous membrane having pore sizes smaller than the mean free path of gas molecules. In this mechanism, the molecules of gas collide with pore walls more frequently rather than colliding with each other hence allowing preferential diffusion of lighter gas molecules through the pores. (2) Gaseous transport through polymeric membrane usually follows solution diffusion mechanism. This mechanism comprises three steps: (i) solubility of preferential permeate at the upstream surface of the membrane; (ii) activated diffusion across the membrane; and (iii) desorption of gas molecules on the downstream side. This mechanism of solution diffusion is carried out by the difference in thermodynamic activities that exist across the membrane as well as by the forces of interaction working between permeating molecules and membrane material as illustrated in Figure 11 below. (3) Zeolites and CMS membranes are common membranes that follow molecular sieving mechanisms. In it, the ratio of the molecular size of gas to micropore diameter typically controls the rate of permeation [94].

## 4. Mixed Matrix Membranes

**Figure 12:**
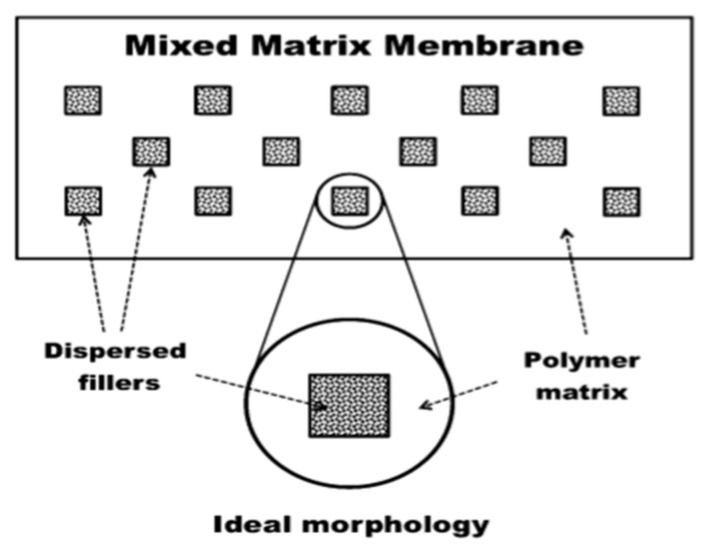
Schematic diagram of ideal MMM morphology [100]. Reprinted/adapted with permission from Ref. [100]. Copyright 2022, IntechOpen.

A lot of research has been conducted to resolve the problems suffered by both inorganic and polymeric membranes. The deficiencies endured by both inorganic and polymeric membranes drove the researchers to create an advanced membrane material that possesses improved mechanical strength as well as higher separation performance and is also economically feasible. Mixed matrix membranes (MMM) which area combination of inorganic and organic materials were then put forward as an idea to achieve enhanced gas separation performance at a low cost. MMM fabrication is considered to be a favorable technology because this composite material enhances mechanical properties as well as amalgamates the superior separation characteristics and stability of inorganic material along with the efficient processability of polymeric material [97,98]. In MMM, inorganic fillers in the form of solid, liquid or both solid and liquid are dispersed in the polymer matrix. MMM combines the advantages of higher selectivity of dispersed fillers along with enhanced processability and mechanical strength of polymers [99]. The ideal morphology of MMM is shown in Figure 12 below.

The white part in the figure above exhibits the continuous phase of the polymeric matrix and the dispersed phase (fillers) is described by small, dotted squares. After the novel review on MMM by Okumus et al. [101], many reports on the likelihood of MMM have been published. The polymer matrix is the continuous phase of the membrane and because of the better process ability of the polymeric material it can be forged into an asymmetric or symmetric, hollow fiber or flat sheet structure. Membranes that are to be used for the purpose of gas separation should be dense and thin in order to provide high permeability and selectivity. Across the polymer matrix, the gas component is transported by following the solution diffusion mechanism. Fillers usually change the permeation properties of the matrix depending on their size, surface chemistry, porosity, and the quantity that is to be added to the matrix. Fillers which constitute the dispersed phases in MMM can either be inorganic, organic or both. To allow molecular sieving mechanism into the matrix, fillers having specific pores can be utilized whereas non-porous fillers improve the gas permeation effects by alteration of polymer chain packing and also by enhancing the free volume or by creating nanogaps in the area surrounded by filler surface [102,103]. Moreover, adsorptive fillers provide the mechanism of facilitated transport by acting as a carrier for a specific gas component. For example, bipyridine-based UiO-67 MOF enhances the selectivity as well as the permeability of CO_2_ as it consists of Lewis basic sites which work as carriers of CO_2_ [104]. Therefore, nanofillers have a major role in altering MMM and enhancing its permeability and selectivity [105]. Many types of fillers have been tested to find the best combination of polymer and filler that possesses good compatibility with each other. Commonly used fillers include silica, metal oxides, zeolites, carbon nanotubes (CNT), graphene oxide and graphene, carbon molecular sieves (CMS), Metal-Organic Frameworks (MOF), etc.

### 4.1. Incorporation of Different Fillers in Fabrication of MMMs

In the fabrication of MMM, solid polymer MMMs have received the most attention. In this type of MMM, both zeolitic and non-zeolitic inorganic fillers can be incorporated into the polymer matrix [105]. Silico-alumino-phosphate (SAPO) and Alumino phosphate (ALPO) molecular sieves are zeolitic inorganic particles and are considered conventional zeolites. Because of their thermal stability and their permeation performances, these micro-porous materials are used in the preparation of MMM for the purpose of gas separation [55]. Zeolitic crystals properties such as specific adsorption and shape selectivity can enhance the selectivity and permeability of polymer films when combined with the process ability of polymer matrix for the purpose of gas separation of various gas pairs. As a polymer matrix both rubbery and glassy polymers were used in the fabrication of zeolitic MMM. The interaction between rubbery polymers and the zeolitic fillers is brilliant because of polymeric chain’s high mobility. In spite of the fact that they exhibit good permeation properties and high mechanical strength, MMM that are fabricated using zeolites and glassy polymers end up having interfacial voids and defects. To eliminate the problem related to unselective gaps mostly occurring on polymer and to resolve the issue related to adhesion; surface modifications are introduced that include the coating of a diluted solution of highly permeable silicone rubber [106]. A plasticizer is added to reduce the intrinsic gas separation performance of polymers [107]. Amine coupling agents and saline is mostly used to enhance both gas selectivity and interfacial adhesion by modifying zeolites’ surface properties from hydrophilic to hydrophobic [108]. It was seen that the selectivity of CO_2_/CH_4_ decreased up to 80% by the embodiment of unmodified zeolites due to unselective void formation [109]. Whereas by the incorporation of modified zeolites, CH_4_/CO_2_ selectivity has boosted 50% as compared to neat membrane. By the absence of unselective voids after modification of the surface, CH_4_ followed a longer permeation path, whereas CO_2_ can easily access through the filler and hence selectivity of CH_4_/CO_2_ improved. Till now many reports have been patented on zeolitic-based MMM as a better alternative to polymeric and inorganic membranes [110].

During the process of gas separation, plasticization is another important phenomenon [111]. At an elevated feed pressure, CO_2_ plasticizes a broad range of glassy polymers [112]. Reduction in the interaction between filler and polymer can cause the plasticization of glassy polymers and declines the performance of membranes in gas separation applications. The CO_2_ also causes an increase in polymer chain segmental mobility, and hence, increases diffusion coefficients of all the penetrants within the membrane. In order to improve plasticization resistance, modification and cross-linking methods were applied widely [113]. The process of cross-linking causes the reduction in polymeric chain mobility by improvement in the adhesion between inorganic filler and polymer. By overcoming the plasticization induced by CO_2_, long-term stability and gas separation performance can be achieved. To obtain a defect-free membrane with better separation performance, priming and sonication technique because of their simplicity is also adapted in the fabrication of MMM. The particle size of filler, besides sonication also helps in better filler dispersion throughout the polymer matrix. Filler particle size that is smaller than 50 nm gives more polymer filler interfacial area and hence enhances the interfacial contact between them [114]. Speaking theoretically, if the particle size of the filler is decreased from R1 to R2, the number of filler particles as well as the available total external area increases by a factor of (R_1_/R_2_) and by (R_1_/R_2_)^3^ [115]. Liu et al. [116] showed that for better dispersion of filler as well as for enhanced gas separation performance it is important and crucial to have a uniform and small filler particle size. Moreover, to avoid particle agglomeration at a high loading of filler and to enhance polymer filler compatibility, functionalization and chemical modifications can be carried out. Fillers that are amine-functionalized provide active sites for nucleophilic reaction and increase the membrane’s solubility coefficient [117]. Moreover, for the fabrication of mixed matrix membranes, the process of silylation is applied on several inorganic fillers as it is considered the most facile modification technique. This process of modification improves compatibility between both inorganic and organic phases as the polymer chains can be attached to the surface of filler through silane bridges [118].

Non-zeolitic inorganic fillers have also received much attention in the development journey of MMM. Metal oxide nanoparticles, carbon molecular sieves (CMS), and porous and non-porous silica nanoparticles are some groups of non-zeolitic fillers. CMS nanoparticles having micro pores are incorporated in the fabrication of MMM as they exhibit remarkable permeation behavior and high productivity [119]. They also exhibit a good affinity to glassy polymers ensuring good contact at the interface. However, in order to prevent the formation of interfacial voids and defects and to enhance selectivity as well as permeability many improvements are being applied in CMS-based MMM [120]. Before undergoing pyrolysis at a temperature of 800 °C for 2 h in a vacuum, CMS was made by using dense matrimid 5218 as a precursor. With the 200 selectivity of CO_2_/CH_4_, and 43.5 barrer permeability of CO_2_, the resulting MMM with CMS exhibited favorable properties for separation. The improvement in separation properties was due to the incorporation of CMS into the polymer matrix.

In order to fabricate heterogeneous Mixed Matrix membranes by a sol-gel process, porous and non-porous nanoparticles of silica in forms such as ceramic, tetraethoxysilane, organosilicate, fumed or colloidal silica is usually dispersed in the polymer matrix [121]. Because of the weak permeability or intrinsic impermeability of silica particles, the addition of these particles in the polymer matrix improves both selectivity and permeability by altering polymeric chains’ molecular packing [122]. MMM, which exhibits an increase in polymer volume without the formation of non-selective voids, has decreased selectivity but better permeation properties. Chemical modifications are expected to eliminate the formation of voids and are carried out with silane coupling agents containing hydroxyl or organo-functional groups [123]. Another form of silica particles, i.e., mesoporous materials is used as filler to enhance the interaction between filler and polymer by penetration of mesopores through the polymer chain. Because of their large surface area, the addition of mesoporous materials in the polymer matrix can bridge polymeric chains through hydrogen bonding and no increase in selectivity was observed because diffusion of gas in mesopores is non-selective [124]. Many approaches have been put forward to increase the selectivity such as creating mesopores in pure zeolites [125], the incorporation of micropores in mesoporous materials [126] or interface composition modification by functionalizing with organic groups, i.e., PMOs (Periodic Mesoporous Organosilica) [127].

A rapid increase in publications has been observed directing towards the applications of the above-mentioned fillers used in the fabrication of solid-polymer MMM. In spite of this, many alternative fillers such as layered silicates, graphene, carbon nanotubes (CNT), metal-organic frameworks (MOFs) have been studied as a new material for fillers having several attractive properties. The remarkable smoothness of the potential energy surface of CNT permits fast diffusion of gas molecules across their channels which in turn results in higher gas permeability without altering the selectivity. Some disadvantages of CNT that restricts its use in the MMM domain include high cost for production, inadequate adhesion between polymer matrix and particles of CNT, as well as entanglement and agglomeration of CNT particles. To improve the compatibility of CNT particles with polymer matrix, surface modifications that are carried out by acid treatments with coupling agents having hydroxyl or carboxyl group is considered to be a frequently used functionalization method. Acid treatment helps in better dispersion of CNT particles in polymer matrix and aids in higher gas molecular diffusivity by opening up the closed ends of CNT [128,129]. Graphene, because of its remarkable thermal, structural, mechanical and electrical properties, has been used as a feasible and cost-effective alternative for CNT in MMM as it belongs to a new class of carbon nanomaterial [130,131,132,133].

Metal-organic frameworks (MOFs) have established a new approach towards the idea of solid fillers in the fabrication of MMM and have evolved as a key material for gas separation, storage and adsorption [134,135]. This new category of porous and crystalline materials that are made by self-assembly of complex subunits having transition metal centers that are connected by several organic ligands (polyfunctional) to create 1, 2, 3-dimensional structures, has gained quite an attention in the past few years. Compelling properties of these hybrid materials include large surface area, low density, high porosity, regularity in framework, high micropore volume, flexible chemical composition because of the existence of strong chemical bond and organic linking units that can be modified [136], adjustable pore size as well as high metal content that provides useful active sites [137]. MOFs, because of their large pore volume and surface area, provide an advantage over other porous materials such as zeolite and activated carbon. The large surface area provides greater contact with targeted species which in turn increases particle effectiveness. Their incorporation in MMM is beneficial as they exhibit better compatibility with polymer matrix because the organic linkers that are present in its structure have strong interaction with polymer chains [138,139]. However, pristine MOF membranes often are unable to attain the required higher selectivities due to imperfections, such as cracks and pinholes, hence their dispersion in the polymeric matrix is mostly preferred [140]. For MMM fabrications, many important aspects should be considered such as good interfacial adhesion among two phases, to avoid the formation of non-selective interfacial voids and also, the diffusion of gas into pores of filler should not be blocked [141]. Several MMMs have been examined previously, based upon a different variety of MOFs and polymers [142,143,144,145,146] and also, several reviews are available already on this topic [10,146,147]. The addition of many different varieties of MOFs such as Bio-MOF-1 [147], MIL-53 (Al) [148], MIL-101 (Cr) [149] and Cu_3_ (BTC)_2_ [150] have produced beneficial results regarding gas permeabilities as well as selectivities.

Zeolite imidazolate frameworks (ZIFs) belong to the class of MOFs and possess excellent thermal, chemical and hydrothermal stability up to 400 °C [151]. One of the most alluring characteristics of MOFs is their potential for adsorption. The large surface area of MOFs having open metal sites behaves as a huge platform for selective gas adsorption [152]. The adsorption capacity of MOFs increases while maintaining their structure at high temperatures because of the presence of structural flexibility [153]. It was reported that at elevated pressure, the adsorption capacity of ZIF-69 improves up to 40% when changing to larger pores [154] and every liter of ZIF-69 is capable of retaining 82.6 L of CO_2_ [155]. In recent years, the uses of MOFs in gas separation applications have been reported. Like all MOFs, ZIFs are able to provide a broad range of configurations and that could be obtained by altering imidazolate linkers and coordination metal. This attribute in turn leads towards different dimensions and topologies of pores. ZIF-8 possessing molecular sieving attributes [156] is a prominent ZIF family member and is commercially available. Hence, ZIF-8 is considered as potentially favorable filler for enhancing membrane properties.

When compared to the other applications, the applications of (MOFs) metal-organic frameworks for the separation of gases are still not very much developed. Despite the fact that MOFs possess many advantageous properties, it belongs to a new class of materials. Understanding of several factors including material cost as well as the influence of different vapors and gases is important prior to its use in industrial applications [157]. In comparison to CMS and zeolites, MOF has no dead volume, low desorption energy, higher BET surface area and high uptake capacity. Within its structure, the existence of an organic linker provides a good interaction with polymer matrices that helps in reducing interfacial defects. With high chemical, mechanical and thermal stability, robustness, and moderate cost, MMM’s development is surely an interesting approach. The heart of the development of MMM is the selection of fillers that are compatible with the polymer matrix and improves the performance of membrane exceeding Robeson upper bound. Table 5 and Table 6 below show the gas separation performance of MMMs in comparison to pristine polymeric membranes.

#### 4.1.1. Zeolite Immidazolate Frameworks (ZIFs) as a Promising Filler for MMMs Fabrication

ZIFs belong to the class of Metal-Organic frameworks and are caged compounds formed by self-assembling of molecules, in which divalent cations, i.e., Zn or CO are coordinated tetrahedrally and are linked by immidazolate anions to form topologies that are similar to those of aluminosilicates zeolites [173]. ZIFs have several advantages over traditional porous materials such as diversified structure, adjustable and porous pore channels, high specific surface area, easy functionalization as well as unsaturated sites and are viewed as next-generation membrane materials [174]. ZIFs possess high selectivity and have the ability to adsorb CO_2_ 82.6 times more than their own volume [155] due to the powerful adsorption of nitrogen-containing groups present in their structure, and it consequently gained significant research attention.

Because of the strong metal-ligand interaction, it is able to retain its structure even at high pressure and temperature owing to its hardness strength and high elastic modulus. The high physical density of ZIFs provides it stiffness to endure high loads hence enhancing its mechanical stability [155]. ZIFs are seen as promising materials for gas storage and adsorption and because of their porous and thermally stable structure they can resist high temperatures up to 600 °C before their structure collapses to form metal oxide. ZIFs exhibit flexibility in their framework with respect to gas adsorptions and are stable under harsh conditions. The benefit to incorporate ZIFs over other nanoparticles is the organic components of ZIF which may help to improve compatibilities between polymer and filler [175,176]. Good gas selective properties of adsorption are shown by ZIF-7, ZIF-L, ZIF-22, ZIF-8, ZIF-69, ZIF-108, ZIF-90 and ZIF-68 because of their special diffusion pathways for guest molecules and narrow pore size distribution [177]. Its capabilities for gas storage and adsorption have been extensively studied for CO_2_ and H_2_ [178]. Even at low pressure, it has high CO_2_ affinity which is associated with the interaction among quadrupole moments of CO_2_ with open metal sites and polar functional groups in ZIFs [155]. ZIFs comprise the ability to separate CO_2_ through a molecular sieving mechanism. Improved molecular exchange, as well as storage, is expected from the small aperture and large cages as compared to MOFs having a straight tubular channel.

Various methods, such as microwave-assisted solvothermal [179], solvothermal at high temperature [152,180,181], accelerated aging [180], thermo-chemical [181] and ultrasound [182] are used to synthesize ZIFs in organic solvents. The usage of flammable and expensive organic solvents such as methanol (CH_3_OH), *N*, *N*-diethylformamide (DEF) and *N*, *N*-dimethylformamide (DMF) in synthesis medium have resulted in being harmful to the environment because of toxicity present in their nature [152,156]. To reduce environmental impacts and the usage of organic solvents, a lot of research has been conductedin order to develop an economical and green synthesis process for the production of ZIF. For CO_2_ capture, ZIFs have been researched broadly as membrane materials and adsorbents as they helps in reducing the rising level of CO_2_ in atmosphere caused from industrial emissions [183].

#### 4.1.2. Zeolite Immidazolate Frameworks-8 (ZIF-8)

**Figure 13:**
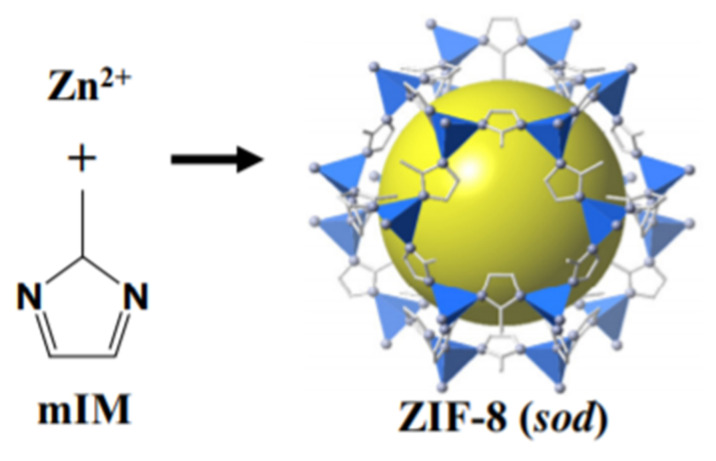
ZIF-8 crystal structure [187]. Reprinted/adapted with permission from Ref. [187]. Copyright 2022, Elsevier.

ZIF-8 is one of the most explored Metal-Organic Frameworks. It possesses a porous crystalline structure having M-Im-M angle that is about 145° and usually coincides with Si-O-Si angle present in many zeolites having a large pore size of 11.6 A° and 6-ring window aperture of 3.4 A° as shown in Figure 13. It possesses good chemical stability against non-polar and polar solvents [151], has high mechanical and thermal stability [184] and has the ability of reorientation of its structure at elevated pressure [185]. Studies regarding the chemical stability of ZIF-8 were conducted by immersing the prepared ZIF-8 in numerous non-polar and polar solvents at different temperatures for a particular time period. ZIF-8 rigid structure has shown strong resistance towards many solvents at high temperatures, for above 7 days [144]. The structure of MOFs generally tends to collapse even at 50 °C in water, this shows its poor stability in water. However, ZIF-8 is able to maintain its structure in water even after 7 days at a temperature of 100 °C, hence exhibiting the excellent stability [186]. Strong bonding among Zn^+2^ and organic linkers, as well as hydrophobic pores, are the reason behind its strong chemical stability [151]. High thermal stability up to 600 °C without damaging its structure is shown by it under an inert environment [151]. Due to the high hardness strength and elastic modulus, ZIF-8 has the ability to retain its structure even at high pressure [184]. ZIF-8′s high mechanical strength is attributed to its stiffness and high physical density at higher pressure up to 100 bar and load without collapsing the structure [187]. ZIF-8 has a high adsorption capacity, a large surface area of 1900 m^2^/g and can be easily synthesized. Another remarkable property of ZIF-8 is its crystal size controllability. Its crystal size is mostly controlled by solvent type, synthesis temperature, rate of mixing, base type additive and the ratio of metal salt-ligand-solvent [188]. ZIF materials were first synthesized by Yichang et al. [189] in an aqueous solution. The process of synthesis was carried out at room temperature and generally took quite a few minutes rather than hours or days in case of non-aqueous conditions [189]. The product obtained was ZIF-8 nano-crystals possessing ~85 nm size and exhibited remarkable thermal, solvothermal and hydrothermal stabilities. As mentioned, ZIF-8 exhibited remarkable chemical and thermal stabilities as compared to other MOFs materials. So, it has managed to gain more attention in applications such as gas separation and storage [190,191,192], chemical sensors [193] and catalysis [194].

#### 4.1.3. ZIF-8 Based Filler in Fabrication of Gas Separation MMM

The development of defect-free MMM is considered challenging as its fabrication suffers from polymer filler incompatibility. Poor interaction between polymer and filler that leads to the formation of non-selective voids can cause pore blockage, polymer rigidification and sieve-in-cage morphology, which deteriorates membrane performance [161]. Thus, having good polymer-filler compatibility is important. In general, utilizing MOF as filler in MMM is reported due to the good interaction among its organic linkers and the polymer matrices. ZIF-8 belongs to the class of MOFs and because of its high stability and exceptional CO_2_ adsorption properties, it can be used as a membrane material as well as an adsorbent for purpose of gas separation [144,195]. ZIF-8 possesses two remarkable advantages. First, it can be easily fabricated and modified as well as its mechanical stability provides substantial scope for application [155]. Second, it provides a sieving window for CO_2_ separation because of its crystallographic pore size which is 3.4 A° and it lies between the pores sizes of CH_4_ (3.8 A°), CO_2_ (3.3 A°) and N_2_ (3.64 A°). Because of these reasons, ZIF-8 is considered one of the most important filler materials in CO_2_ separation [196]. The filler size in the fabrication of MMM greatly affects the performance of the membrane [197]. A small particle size provides free volume through better polymer chain disruption and also increases polymer filler interface [198]. Studies show that incorporating nanofiller even at low loadings can significantly improve membrane separation properties. As compared to the smaller particle size, the larger filler particle size offers less particle number per unit area at the same mass load, hence, providing less opportunity for interaction with the polymer matrix. As an expensive organic solvent is required to synthesize ZIF, its utilization as filler is bounded by the high cost. Researchers are more concerned to develop new kinds of ZIFs rather than making it cost effective as most of the ZIFs are still in their early stage of development.

Studies conducted by Ordonez et al. [144] on ZIF-8 revealed a huge increase in selectivity of CH_4_/CO_2,_ i.e., around 300% better than neat Matrimid membrane [144]. The betterment in the performance of the membrane was attributed to the ZIF-8 molecular sieving mechanism dominant at high loading. Therefore, the permeation of CH_4_ was restricted. Interestingly, as per the author’s report, ZIF-8 agglomeration was observed even at 60 wt% loading, the selectivity of CH_4_/CO_2_ of the produced mixed matrix membrane was twice that in comparison to the neat membrane. Without the decline in the performance of the membrane, compatibility of Matrimid with ZIF-8 allowed higher loading of filler. As the loading of ZIF-8 increased up to 80 wt%, the membrane became fragile. Zhang et al. [199] demonstrated that surface modification of ZIF-8 crystals by thermal treatment in H_2_ and N_2_ atmosphere and ammonia impregnation can increase the amount of basic sites on the surfaces of samples and hence results in an improvement in selectivity and adsorption capacity towards CO_2_ [199].

### 4.2. Separation Performance of Mixed Matrix Membranes

Porous solids having greater permeability as well as selectivity than polymers are mostly preferred as fillers in MMMs. As discussed earlier, many different filler particles including MOFs, zeolites, CMS, and other nanoparticles were incorporated into MMMs to enhance their gas separation performance. Interfacial interactions among these porous particles and polymer such as chain rigidification, pore blockage, and increase in free volume, as well as the formation of interfacial voids usually control gas transport through MMMs [107,200].

Generally, permeability is described as the product of solubility (*S*) and diffusivity (*D*). In MMMs, the variations in permeability can be described by utilizing solubility and diffusion coefficient. The influence of polymer-free volume on the diffusion coefficient of penetrants can be explained by employing the statistical mechanical hard-sphere model of diffusion in liquids that were put forward by Cohen and Turnbull. The diffusion coefficients of penetrants (*D*) can be explained by employing the following Equation (1).

(1) $$D=Ae^{\left(\frac{-\gamma V^{*}}{Vf}\right)}\;$$

*A* = pre-exponential factor, i.e., weakly dependent upon temperature; V* = minimum free volume element size that could accommodate penetrant molecules; γ = overlap factor introduced in order to prevent double counting free volume; *Vf* = average free volume in media that is accessible to transport of penetrants.

According to the above equation, it is expected that an increase in free volume can enhance the diffusion of penetrants. The free volume that was measured by utilizing PALS (positron annihilation lifetime spectroscopy) in PTMSP/FS (Poly(1-trimethylsilyl-1-propyne)/Fumed silica) MMM displayed enhancement in free volume with the loading of FS and correspondingly substantial increase in permeability of N_2_ was noticed with an increase in loading of FS [201,202]. The penetrant’s solubility coefficient is dependent upon the interaction between filler and polymer. The functional groups of filler and polymer such as hydroxyl amine interact with polar gases such as SO_2_ and CO_2_, and this results in an increase in solubility of penetrants in MMMs which in turn increases gas permeability. The penetrant’s solubility dependence with enthalpy of sorption and temperature is narrated in terms of van’t Hoff relation (Equation (2)).

(2) $$S=S_{0\;}e^{\left(\frac{\Delta Hs}{RT}\right)}$$

Δ*Hs* = enthalpy of sorption; $S_{0}$ = constant; R= ideal gas constant; *T* = absolute temperature.

An increase in interaction among penetrant molecules and functional groups decreases Δ*Hs*, and as a result the solubility of gas increases. Within poly(amide-6-b-ethylene oxide) and silica MMM, an increase in silica loading resulted in an increase in CO_2_ solubility coefficient. It was because of the strong interaction of molecules of CO_2_ with SiO_2_ as well as with polyimide block in PEBAX [203].

## 5. Hollow Fiber Membrane Configuration

Industrial applications involving membranes require thousands of square meters in order to execute a gas separation process at a large scale. Hence, the process of membrane separation should be efficient and economical if it is to be used commercially. Membrane configurations typically refer to the geometry of the membrane and its position in connection with the flow of feed and permeate. Moreover, it determines the pattern in which membranes are packed in modules. Membrane modules are actually a choice of configuration in which various formats including hollow fibers, spiral wound, plate and frame and tubular membrane modules are available. Hollow fiber (HF) membrane modules are preferred in industries because of their self-supporting ability, easy handling in module construction, larger surface area per unit volume as well as good gas separation abilities [204].

**Figure 14:**
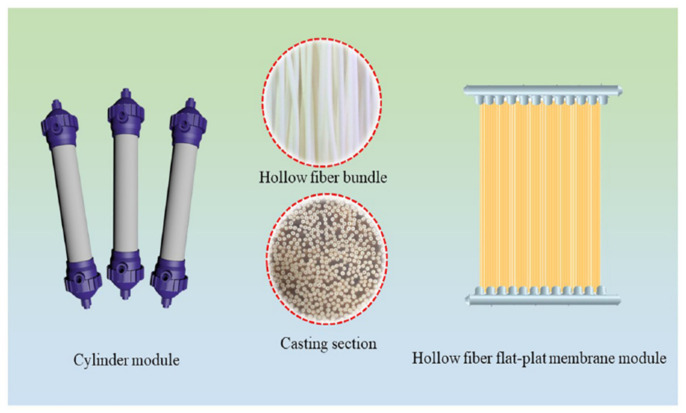
Commercial HF membrane module type [210]. Reprinted/adapted with permission from Ref. [210]. Copyright 2022, Elsevier.

The concept of hollow fiber (HF) membranes and their modules prepared from polymeric materials was first introduced by Mahon almost 50 years ago in his patents [205]. Hollow fiber membranes as compared to flat sheet and inorganic membranes are considered a better choice for membrane modules because of advantages such as good flexibility, large surface areas and their self-supporting property [206,207]. To use a flat sheet membrane module in filtration applications, complex hardware such as spacers and porous supports are required. Good flexibility, as well as the self-supporting quality present in hollow fiber membranes, minimizes the complexity in hardware fabrication amid module assembly and operation. HF membrane modules can be fabricated by HF membrane bundles consisting of a large number of HF membranes and are normally in form of HF flat plate membrane modules and in cylinder modules. High productivity is achieved because of its high packing density and large surface area and also HF membrane provides high energy efficiency in obtaining complete mixing in modules. Figure 14 below shows the type of module for commercial HF membrane. The performance of HF membranes is determined by the pore size and its distribution which controls the selectivity, also by the properties of the membrane material which governs the selectivity and intrinsic permeability, and by the thickness of the selective skin layer which controls the membrane flux [208,209].

Chung and Kafchinski proposed that the formation of hollow fiber membrane is also controlled by rheological properties of spinning dope, the flow rates of bore fluid, properties of external coagulant, dope and bore fluid flow rates, temperature and also shear stress within spinnerets’ annular orifice [211]. The parameters that control the fabrication of flat sheet membrane are different from the ones that control the fabrication of HF membrane. For the formation of the asymmetric flat sheet membrane, only one coagulation surface is required, whereas two coagulations, external and internal are used in the spinning process of HF. Moreover, for the flat sheet membranes, a waiting period is needed before dipping them in the coagulant bath, but the coagulation starts instantly in HF fabrication after extrusion from spinneret [212,213,214]. Moreover, as compared to the flat sheet membranes the spinning dope for HF has high elasticity and viscosity hence the development of macrovoid in the HF spinning process is more complicated [215]. The ratio of surface area to volume in HF modules is 30 to 50 times greater than the spiral wound modules, which is almost 10,000 m^2^/m^3^ [216]. The tubular structure of hollow fiber can bear high-pressure differences up to 1000 psi [217]. HF membrane’s unique configuration provides them with exceptional mass transfer properties and due to this, they can be used in various commercial fields of applications such as medical field (dialysis), gas separation, water treatment, food processing, and azeotropic mixture separation [218,219,220].

Membranes can be fabricated by stretching (porous membranes), nucleation-track-etching (membranes with cylindrical pores), melt-extrusion (dense membranes), particles-sintering (porous membrane), template-leaching (porous membranes), solution coating, phase inversion (asymmetric membranes) and swelling a dense film (porous membranes), etc. Most of the polymeric membranes are asymmetric, formed by the method of phase inversion and are available commercially. Many studies have been performed on the fabrication and characterization, as well as on the applications of HF membranes over the last few years and among them, polysulfone (PSF), poly vinylidene fluoride (PVDF), poly tetrafluoroethylene (PTFE), poly ethersulfone (PES), poly acrylonitrile (PAN) are membrane materials that are mostly used. When the material used to fabricate the membrane is selected, the mechanical strength and permeability of the membrane are determined by its structure, which mostly depends on the process and technique used to fabricate the membrane. Membrane’s mechanical stability especially long-term robustness and resistance towards chemical cleaning processes are some of the important factors alongside permeability and selectivity. HF membranes that are prepared by immersion precipitation technique normally have high permeability but low mechanical stability because of the loose support layer and dense layer. Therefore, hollow fiber membranes can easily get damaged or broken frequently in commercial applications by high pressure, frequent chemical cleaning, and airflow. Hence, HF membranes having exceptional mechanical strength and adequate separation properties are essentially needed.

In order to increase the mechanical strength and properties of hollow fiber membranes, many studies have been performed. The use of high strength tubular braid that is coated with a separation layer is one of the techniques that is found to be effective [14]. After the invention of the braid reinforced hollow fiber membrane by Hyano et al. [17], many patents are accepted but still very limited data is available in the open research literature. On the use of braid reinforced hollow fiber membrane, there were only 18 studies available in literature by August 2019. Liu et al. [14] were the ones who did the earliest studies on the braid reinforced hollow fiber (BRHF) membrane in which the effect of filament numbers on the mechanical endurance of membrane was examined [14]. Studies focusing on the use of BRHF membrane were performed by more research groups after 2014. The remaining published studies focused on utilizing spinning systems that permit the use of braid. Studies generally include finding out the effects of the braid, as well as polymer types on the interrelationship between separation and support layer. BRHF membranes are normally used in MF (microfiltration), UF (ultrafiltration) as well as in MBR (membrane bioreactors) processes. As they have high mechanical endurance in applications in which high pressure is involved, they can also be utilized in NF (nanofiltration), RO (reverse osmosis) and in gas separation technologies. Reinforced hollow fiberultrafiltration (UF) membranes were used by Sengur-Tasdemir et al. [221] to make NF membrane by utilizing protein (Aquaporin Z) on the interfacial polymerization layer [221]. The membrane efficiency was characterized by utilizing braid-free TFC (thin film composite) membrane on a comparative basis. On comparison with braid-free thin film composite membranes, both reinforced Aquaporin Z and reinforced thin-film composite membranes had high permeability of water and the same rejection performance.

## 6. Potentiality of Braid Support Hollow Fiber Membrane for Use in Gas Separation Applications in Future

A membrane should have adequate mechanical strength, high permeability and good chemical stability. The chemical stability of the membrane is generally determined by the membrane material’s chemical composition. While, the permeability and mechanical strength of the membrane are dependent on the structure of the membrane, which is determined by the process used to fabricate the membrane [222]. Mechanical strength and robustness are important factors in terms of the fabrication of membranes along with permeability and selectivity. Hollow fiber membranes have a higher specific surface area when compared with flat sheet membranes. Moreover, it is easy to assemble hollow fiber membranes into modules for different applications as they are also mechanically self-supporting [223]. Hollow fiber membranes that are fabricated by the immersion precipitation technique have higher permeability and lower mechanical strength due to the loose support layer and dense layer [14]. So, they are prone to damage at high pressure or airflow. Important research is being conducted in order to enhance the mechanical characteristics of HF membrane. Coating a separation layer onto a tubular braid possessing greater mechanical strength is considered a productive approach [224].

The fabrication of reinforced hollow fiber membrane has not yet been extensively analyzed in research-based literature. Cooper et al. [16] first described the concept of fabrication of braid reinforced hollow fiber membrane, in which they outlined the application of embedded braided material and used casting bob to make reinforced fibers [16]. However, this technique was found to be inappropriate for fabricating capillary membranes. The concept of a semi-permeable composite membrane was described by Hanyo et al. [17] which comprises porous material and also fibrous support, which is completely embedded in a porous material wall [17]. In this case, the reinforcing fibrous support is fully embedded in the polymer instead of a polymer coating on it [17]. The concept of the hollow fiber membrane having a tubular macroporous support (particularly braid) was described by Mahedran et al. [225] which is coated with a semi- permeable thin tubular asymmetric polymer film on the outer surface. The voids present in braided material are quite larger than pores within the film yet are smaller enough to permit significant penetration of dope solution to the inner side of braid material. Braid reinforced hollow fiber (BRHF) membranes are generally utilized in ultrafiltration membranes for the treatment of industrial wastewater and are also used for microfiltration processes. They have higher mechanical strength and can be utilized in the process of reverse osmosis for the treatment of drinking water and also in wastewater recovery. For the treatment of low-quality water sources, a reinforced HF nanofiltration module is considered to be a suitable substitute for tubular membrane, as well as spiral wound membrane. When compared with optimized spiral wound module, it is observed that optimized HFNF module would give 100% increased performance [226]. One of the disadvantages of nonreinforced hollow fiber membranes is their low mechanical strength which limits the application of hollow fibers in separations involving high pressure [227]. Braid reinforced HF membrane solves this problem of mechanical strength. Chen et al. [228] fabricated braid reinforced poly(mphenyleneisophthalamide) (PMIA) hollow fiber membranes via a dry wet-spinning process. Favorable interfacial bonding was observed between reinforced braid and separation layer as well as an increase in tensile strength was observed and, the tensile strength of braid reinforced PMIA membranes surpassed 170 MPa [228]. This research study indicated that BRHF membranes possess superior mechanical strength and can tackle high feed pressures [228]. BRHF membranes have succeeded in gaining researchers’ interest and attention because of their low cost and simple fabrication process, efficient separation ability as well as exceptional mechanical strength. This form of hollow fiber membrane has remarkable tensile strength contributing to the membrane’s long life and hence can work efficiently under high-pressure conditions in comparison with non-reinforced hollow fiber membranes. Because of these advantages, it is expected that they can also perform well in high pressure requiring gaseous separations. Yet, further research is needed to test their performance in natural gas separation applications involving high pressures.

### 6.1. Fabrication of Braid Reinforced Hollow Fiber Membrane

**Figure 15:**
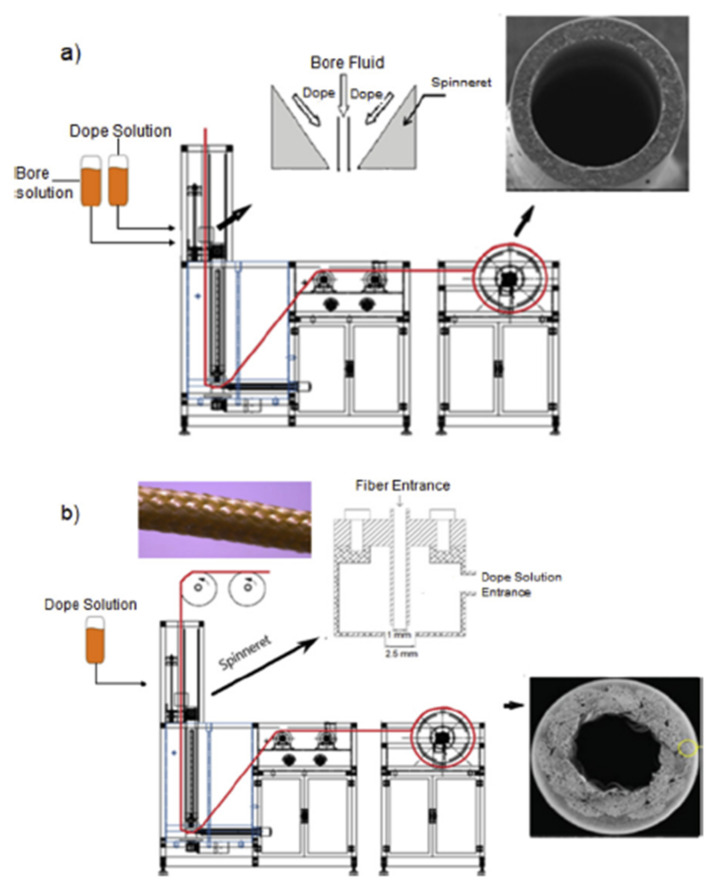
(**a**) spinning line of hollow fiber membrane (**b**) braid reinforced hollow fiber spinning line [15]. Reprinted/adapted with permission from Ref. [15]. Copyright 2022, Elsevier.

The phase inversion method is used for the fabrication of hollow fiber membrane. A typical production line of hollow fiber membrane is shown in Figure 15a below. The openings in the spinneret are available for bore liquid and polymer dope solution to guarantee the shape of the cast is HF.

HF membrane is categorized into reinforced type membrane and a single membrane type. In the reinforced-type of membrane, polymer resinous thin film is coated on a tube shaped braid or fabric while in single membrane type, hollow fiber membrane consists of polymer resin thin film without the utilization of supporting material such as tubular braid/fabrics. A support layer made of Polyethylene terephthalate is normally used as a braid support layer.

The reinforced hollow fiber membrane shows excellent mechanical properties as it uses a tubular braid or fabric as a reinforcing material. Figure 15b shows a production line of braid reinforced hollow fiber membrane. For the production of braid reinforced HF membrane, instead of bore liquid the braid support is passed across the spinneret’s center hole.

### 6.2. Effectof Support Layer Composition on BRHF Membrane

One of the major problems in membrane processes is membrane fouling. Delamination is the membrane peeling off the surface of the braid and can occur due to cleaning of the membrane by back flushing. Out of many membrane materials utilized to fabricate hollow tubular reinforcing braids (e.g., polyimide, polyester, polyethylene, aramid, fiberglass, nylon, etc.), braid composed of fiberglass is more prone to delamination because of poor adherence of membrane to the surface of braided material [222].

The quality of the braided support layer can be determined by many parameters. Braided textile material has many breaks in fibers and is manufactured with standard equipment used for braiding and is made from yarn that is commercially purchasable. Fuzz, which is the build-up of broken fibers, can cause imperfections in polymer film that is coated on the braid’s surface. Whiskers which are splintered filaments stick out from the support layer’s surface and can result in polymer lean layers with pinholes or polymer layers of increased thickness.

Braids such as hybrid, homogenous and heterogeneous are accessed by the interfacial bonding between the tubular braid and separation layer. A braid support hollow fiber membrane was fabricated by Lee et al. [18] in which a thin film of polymer resin was coated on the surface of the reinforcing tubular braid [18]. Zenon Environmental Inc fabricated a reinforced HF membrane that consisted of an asymmetric surface separation layer and braid supported tubular matrix via a process of coating [225]. This BRHF membrane showed superb mechanical strength. Liu et al. [14] researched the fabrication of PET threads reinforced PVDF HF membrane and determined that rupture/tensile strength of the threads reinforced membranes was significantly enhanced by 10 Mpa [14]. Although, problems were faced by this type of membrane as there was weak interfacial bonding between the reinforcement and surface layer and the surface layer was peeled quite easily from the reinforcement because of thermodynamic incompatibility between them, i.e., heterogeneous membrane. Hence, a homogenous reinforced HF membrane was fabricated in which the reinforced layer and surface layer are of same materials and can enhance the interfacial bonding between them. Fabrication and characteristics of homogenous reinforced PVDF hollow fiber membranes were investigated by Zhang et al. [229] and they discovered that there was favorable interfacial bonding between matrix membrane and surface coating layer [229].

**Figure 16:**
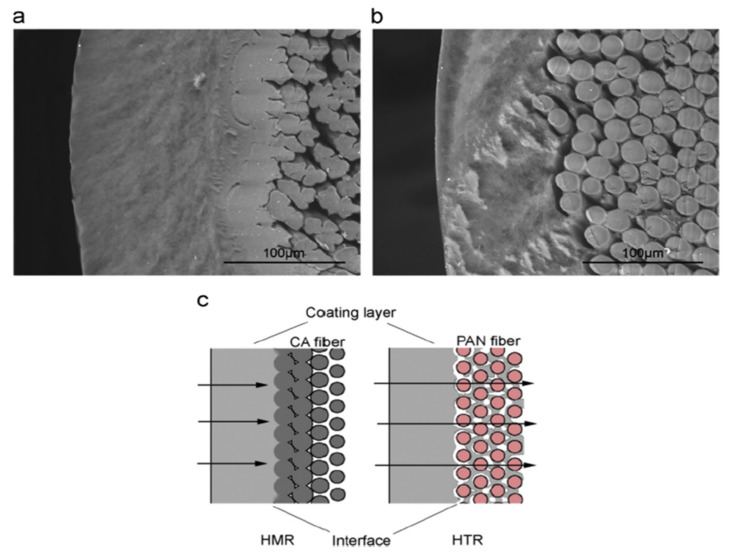
Comparison between interfaces of homogenous and heterogeneous braids: (**a**) HMR interface; (**b**) HTR interface; (**c**) Schematic representation of homogenous and heterogeneous reinforced interface [230]. Reprinted/adapted with permission from Ref. [230]. Copyright 2022, Elsevier.

Fan et al. [230] in another research based on heterogeneous and homogenous braids showed that CA (cellulose acetate) fibers in a braid can swell by a dope solution that results in low pore connectivity (outer to inner surface) as well as a decline in permeation [230]. The gap between the braid and separation layer is observed using polyacrylonitrile fibers as shown in Figure 16 [230]. By combining the advantages and disadvantages of homogenous reinforced membranes, a novel braid which was named as ‘Hybrid braid’ was formulated which is a combination of both HMR and HTR methods. They fabricated braided reinforced cellulose acetate HF membrane via coating hydrophilic polymer solution of cellulose acetate onto a hybrid braid composed of PAN and CA fibers. In hybrid braids, the effect of the PAN/CA ratio on the interfacial bonding was estimated. When compared with pure PAN and CA braid, BR CA membranes on using hybrid braid, i.e., PAN/CA exhibited significant bonding strength as the compatibility or affinity between braid and coating layer as well as the infiltration distance of coating solution could be managed and controlled by changing PAN/CA ratio within hybrid braid [230].

**Figure 17:**
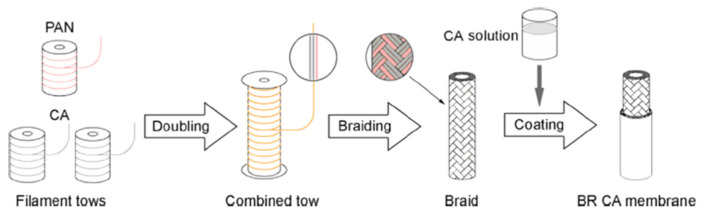
Schematic representation of BR CA membrane preparation process [230]. Reprinted/adapted with permission from Ref. [230]. Copyright 2022, Elsevier.

Two-dimensional braiding technique was used to prepare a hybrid braid consisting of PAN and CA filaments as depicted in Figure 17 [230], this hybrid braid not only bestowed the membrane with favorable interfacial bonding but also controlled the effect of CA fiber swelling on the permeability of the membrane.

**Figure 18:**
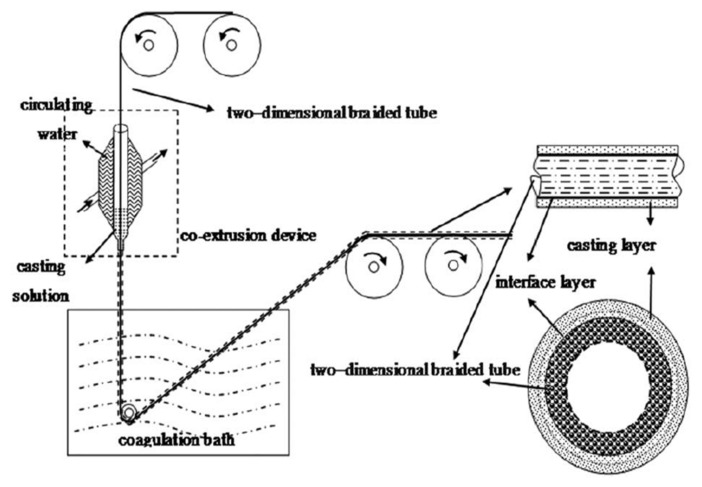
Schematic representation of braided tube reinforced two-dimensional PAN membrane [231]. Reprinted/adapted with permission from Ref. [231]. Copyright 2022, John Wiley and sons.

In a study conducted by Quan et al. [231], homogeneous and heterogeneous braid reinforced PAN HF membranes were fabricated via dry-wet spinning technique as shown in Figure 18 in which PAN polymer solution coating was applied on two dimensional PET and PAN braid surface [231]. Favorable interfacial bonding was observed in the braid reinforced PAN HF membrane between the coating layer and the braid because of the presence of the interface layer. The outer surface of the braid reinforced PAN HF membrane was a dense layer as a separating functional layer. The increase in PAN concentration resulted in a decrease in the maximum pore size of the braid reinforced PAN hollow fiber membrane. The study also showed that the interfacial bonding state of the two-dimensional PAN braided reinforced homogeneous PAN HF membrane was much better and stronger than the two-dimensional PET braided tube reinforced heterogeneous PAN HF membrane. The higher interfacial bonding in BR HF membrane was due to the presence of interfacial layer that was created by coating solution which penetrated into gaps of the braid and formed a part of it, also braid reinforced PAN HF membranes that were fabricated through BR method exhibited remarkable mechanical properties with a tensile strength that was greater than 80 Mpa. However, there was a difference in the amount of coating solutions that infiltered into the gaps of two-dimensional PAN and PET braid, the former was better. The reason behind it was that PAN coating solution and PET two-dimensional braid were thermodynamically incompatible and DMAC (Dimethylacetamide) was not cosolvent of them, hence, there was a poor penetration of PAN solution into two dimensional PET braid. However, two-dimensional PAN braid and PAN casting solutions were compatible thermodynamically and DMAC was a superb cosolvent of them so PAN solutions had excellent penetration into the gaps of two-dimensional PAN braid.

In another study conducted by Zhou et al. [232], BRHF was fabricated by employing an alkaline treated braid and an amphiphillic copolymer/PVC blend via NIPS technique having a mechanically stable hydrophilic coating layer [232]. On the surface of the PET braid more polar groups appeared and after the alkaline treatment, the braid became more hydrophilic. The hydrophilic groups on the surface of the braid, as well as the weight loss of the braid, increase with an increase in treatment time and concentration of alkaline in alkaline treatment and this, in turn, increases the bonding strength between the braid and coating layer as strong polar-polar interaction was created between the hydrophilic coating layer and the braid surface, also polymer coating solution infiltration was also encouraged. This coating layer infiltration brought about improvement in bonding strength. The study showed that when the PET braid was treated with KOH (*Potassium hydroxide*) solution (3 wt%) for about 1 h at 90 °C or KOH solution (1 wt%) for about 6 h, the bonding strength between modified PET (1.1 MPa) braid and hydrophilic coating layer was two times greater than that was between original PET braid (0.6 Mpa) and the coating layer. Hence, this new approach is expected to enhance the bonding strength between the braid and the coating layer without changing membrane properties and it also has potential for operation in membrane engineering.

In a study conducted by Liu et al. [233] PVC BRHF membrane was prepared by employing a dry-wet spinning technique. PVC polymer solution mixture was coated uniformly onto the tubular braid containing PAN and PET fibers, and an investigation of the effect of braid composition on performance and structure of BR PVC HF membrane was carried out. The study indicated that on using PET and PET/PAN hybrid braids as reinforcement, the fabricated BR PVC HF membrane formed two layers containing a separation layer and a tubular braid support layer. However, on using PAN tubular braid as reinforcement, a sandwich structure showed up revealing outer separation layer, inner polymer layer and tubular braid support layer. BR PVC HF membranes that were fabricated by employing PET/PAN hybrid braid exhibited favorable interfacial bonding as compared to the membranes that were fabricated using pure PAN or PET tubular braids. The BR PVC HF membrane that was prepared using PET/PAN hybrid braid exhibited tensile strength that was greater than 50 MPa. With the increase in PAN filaments in PET/PAN hybrid braids both tensile strength and elongation at break decreased.

**Figure 19:**
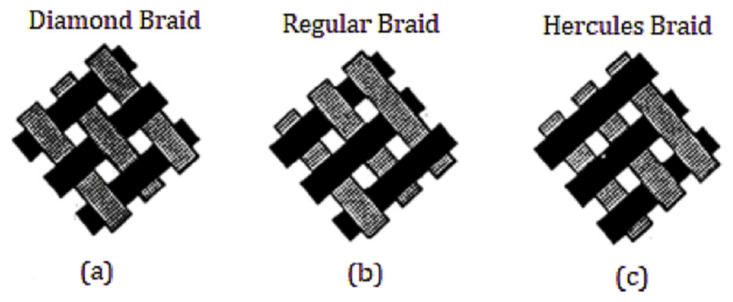
Braid support material with (**a**) Diamond (**b**) Regular and (**c**) Hercules pattern [15]. Reprinted/adapted with permission from Ref. [15]. Copyright 2022, Elsevier.

Different types of braid are shown in Figure 19 below that can be used for BRHF membranes. Braids are categorized as diamond, regular and Hercules braids, based on the interlacement. Diamond braid as shown in Figure 19a has an alternation of one strand advancing above and below other strands. Figure 19b shows a regular braid with alteration of two strands above and below in repeat and Figure 19c shows Hercules braid having a 3 up and 3 down structure. Diamond braid is a popular type of braid and is preferred because it provides extra porosity, elasticity and homogeneity [234]. The regular braid’s porosity is low therefore dope solution between braids causes peeling. The surface roughness is very high in Hercules braid therefore thicker membrane casting is required to fabricate membrane with a smooth surface.

If the support layer’s open weave has a very high porosity, polymer penetrates into the bore of the braid and there will be a sudden reduction in permeability. If the weaning is very tight, it will lead to poor adhesion of polymer and there are higher chances of peeling of the polymeric layer from the braid. The weaning of fiber is an essential parameter for the fabrication of membrane. Fibers that are weaved tightly can foul because of space restriction and, in turn, leads to insufficient moving in order to remain clean or rubbing against each other. On contrary, loose packing of fibers will help fibers in improved repetitive twisting. Cylindricity which is defined as fiber circularity is important. Cylindricity that is less than 0.8 caused uneven thickness of membrane that led to irregular flux as well as defective areas which ended in a film of polymer with undesirable thickness variations leading to variations in flux and defective areas that are rapidly fouled. The cylindricity of the braid should be close to 1.0 [234].

Young’s modulus is another important braid parameter. The aim is to attain high strength by selecting a strong yarn such as aramid, glass and other materials with greater modulus to get an advantage from the material’s stability and high strength. For instance, a braid fabricated from threads of glass multi will have lesser than 5% elongation at break, moreover, these are also considered to be non-shrinkable. High modulus yarns usually impart inadequate film adhesion to the braid surface and are not desirable. If the braid is wet, it will be too weak for prolonged service [234].

### 6.3. Effect of Polymer on BRHF Membrane

The concentration of polymer and additives along with their types regulate the structure of coated film layer. To fabricate the BRHF membrane in which a thin layer of film is coated onto the surface of supporting material or reinforcing material of the tubular braid, thermodynamic stability varies depending upon the dope solution composition that is used for coating. If the dope solution is thermodynamically stable then the predicted cross-sectional structure is mostly finger-like, and if the dope solution is unstable thermodynamically, then there will be no defected regions and the expected structure will be sponge-like.

The dope solution contains polymer, porogen (most probably a hydrophilic additive) and organic solvent for the polymer used. Typical polymer resins are polyethersulfone (PES), polyacrylonitrile (PAN), cellulose acetate (CA) [227], polyimide, polyesterimide, PMIA [228], polysulfone (PS), Polyvinylidene fluoride (PVDF). Similarly for organic solvents common choices are dimethyl formamide (DMF) [235], *N*-methylpyrrolidone (NMP) [236], dimethylacetamide (DMAc) [237]. Commonly used porogen are, polyvinylpyrrolidone (PVP) [238], glycerol and polyethylene glycol (PEG) [239].

To fabricate the braid reinforced hollow fiber membrane Lee et al. [224] utilized PSf [224]. The dope solution was made of PVP (as porogen) 11–19% by weight, PEG that is also used as porogen 10–11%, polysulfone 13–17% by weight and DMF was used as an organic solvent. GO (graphene oxide) between 0.0% and 0.7% was added by Hao et al. [240] in PVDF material for fabrication of braid reinforced hollow fiber membrane for separation of oil-water [240]. Braid was first pre-treated with NaOH and then with distilled water in order to enhance the interfacial bonding. With the increase of graphine oxide amount to 0.5% in polymer matrix resulted in a narrowing membrane’s pore size distribution with increased porosity (38.5% to 47.26%) and increased pore diameter (0.09 nm to 0.16 nm). When the amount of graphene oxide was further increased, it resulted in decreased porosity and pore size. The membrane’s stability came out to be excellent as the performance of membrane (0.5 wt% GO) was not declined even after getting fouled with water/oil mixture after cleaning. Moreover, the effect of cellulose acetate concentration in dope solution for fabrication of BRHF membrane was studied by Fan et al. [230]. The study showed that a high concentration of CA that is greater than 10 wt% created a smooth and dense outer surface and also resulted in an increase in interfacial bonding between the separation layer and braid, as well as resulted in increased bursting and tensile strength [230]. Chen et al. [228] made BRHF membrane by utilizing PMIA polymer and varied the concentration of polymer used in the range of 5–15 wt% and revealed that membranes in which PMIA is used as polymer have better mechanical stabilityas well as with the increase in PMIA concentration, sponge-like structural formation also increased [228].

### 6.4. Influence of Spinneret Design and Spinning Speed on BRHF Membrane

During the fabrication process of braid reinforced HF membrane, the coating of polymer is accomplished by using the unique design of spinneret. Generally, membrane thickness that is obtained outside of the braid is between 0.01 mm and 0.1 mm [241].

**Figure 20:**
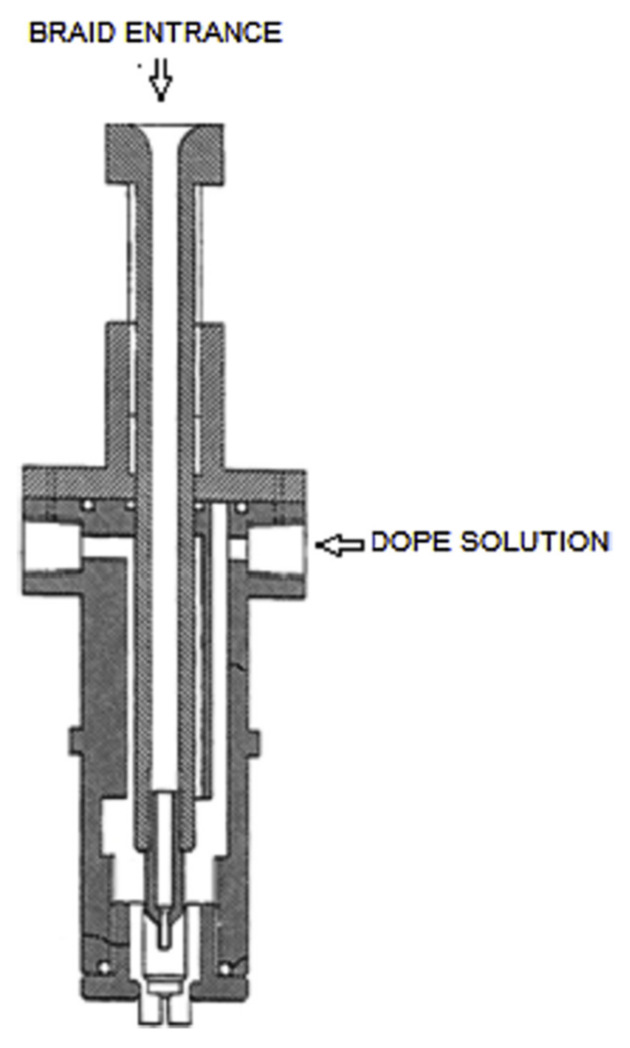
Nozzle design for production of BRHF membrane [15]. Reprinted/adapted with permission from Ref. [15]. Copyright 2022, Elsevier.

Figure 20 shows a unique nozzle design that was used by Mahendran et al. (2002) to fabricate braid reinforced HF membrane [234]. This nozzle has an inner barrel consisting of the internal bore, via which the tubular braid is moved to the nipple’s axial bore which is secured at the inner barrel end. Before coating of the braid with a dope solution, rounding space is provided by the bore to help the braid in acquiring a circular cross-section. The diameter of the rounding orifice is in the range of 1% to 10% of the braid’s nominal diameter. The design of the nozzle controls the dope solution quantity that is flowing through the nozzle, measures the right quantity of dope solution over the surface and dispenses the measured quantity evenly over the surface of the braid. The thickness of the coated layer is dependent upon dope viscosity, braid pulling rate as well as on the dope film thickness that will be coated on the braid prior to its immersion in coagulant.

**Figure 21:**
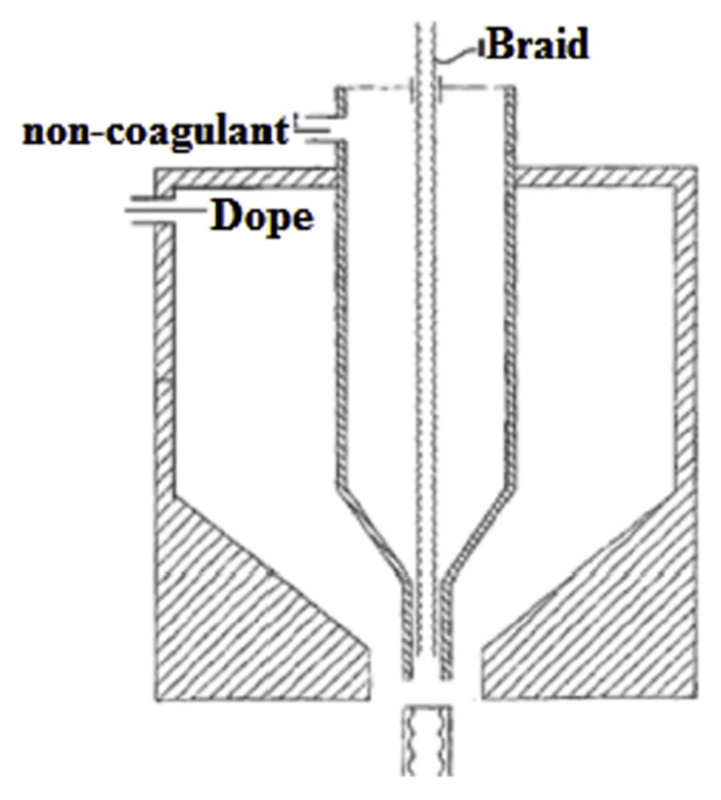
Nozzle design for BRHF membrane production [15]. Reprinted/adapted with permission from Ref. [15]. Copyright 2022, Elsevier.

The second nozzle design type that is utilized to fabricate the capillary membrane is shown in Figure 21. In this type of spinneret, before the braid support (that is entered through the nozzle) encounters dope, a non-coagulant solution is fed through pressure difference across the opening in order to keep the fiber wet. Non-coagulant liquid, dope solution and the braid meet each other at the tip of the nozzle and braided support is then coated by dope solution. The excessive non-coagulant is scraped out by the nozzle leaving behind the liquid non-coagulant only in the inner channel and pores of the braid. The braid with a coating of dope solution on its outer surface and having non-coagulant liquid inside then enters into the water bath. This nozzle design restricts the dope solution from penetrating into the pores of the braid, as well as avoids the anchoring effect of dope coagulating when it meets pore liquid.

**Figure 22:**
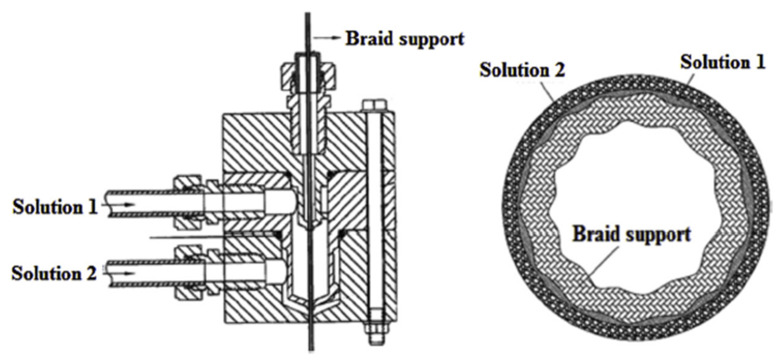
Nozzle having at least 2 different inlets [222]. Adapted from [222].

Another nozzle design is shown in Figure 22 that is used for the fabrication of BRHF membrane. The nozzle is composed of many inlets which coat the support with several layers to obtain a composite membrane with zero defects [222]. This nozzle design guarantees strong chemical bonding and physical adhesion between membrane and tubular support. The stronger binding is because of two techniques: (1) To apply an adherent at the composite membrane’ support side after its formation in order to bond the support and membrane together; (2) by the addition of adhesive permeable layer between tubular support and membrane to bond them together during fabrication of membrane. Hence, the membrane produced can bear high back pressure.

To achieve the predetermined thickness of the separation layer on the reinforcing material’s surface, BRHF membrane’s spinning speed is crucial. The quantity of dope that is introduced in the spinneret, as well as the advancing speed of the tubular braid, must be balanced. The relation between the feed rate of dope solution and the tubular braid’s advancing speed is expressed by Lee et al. (2008) and is defined in Equation (3) [224].

(3) $$Q=D_{o}\pi \rho \vartheta T\;$$

*Q* = Dope solution feed rate (ml/min); ρ = dope solution density; ϑ = braid advancing speed; *D_o_* = braid outer diameter; *T* = dope solution thickness.

As mentioned above, the relationship between the advancing speed of tubular braid and dope solution feed rate is revealed by Lee et al. [224]. According to it, thinner coating layer formation is normally expected when the braid’s advancing speed is high. If the braid’s advancing speed is very high than the spinning dope’s feed rate, some braid parts might not be uniformly coated by dope solution. If the braid’s advancing speed is lower, non-uniform and irregular membrane having a partially thicker coating layer is normally expected. Hence, advancing speed, dope solution density and feed rate must be optimized in order to fabricate the membrane having a uniform thickness.

The most appropriate coating is obtained when K is in the range of 200–300 g/m^2^. Greater values of *K* lead to the formation of a thick coating layer (Equation(4)) [224].

(4) $$k(g/m^{2})=\frac{Q\left(g/\min\right)}{v\left(m/\min\right)D_{o}\left(m\right)}\;$$

Table 7 below lists some production parameters and their effects that are involved in BRHF fabrication.

### 6.5. BRHF Membrane Morphology

**Figure 23:**
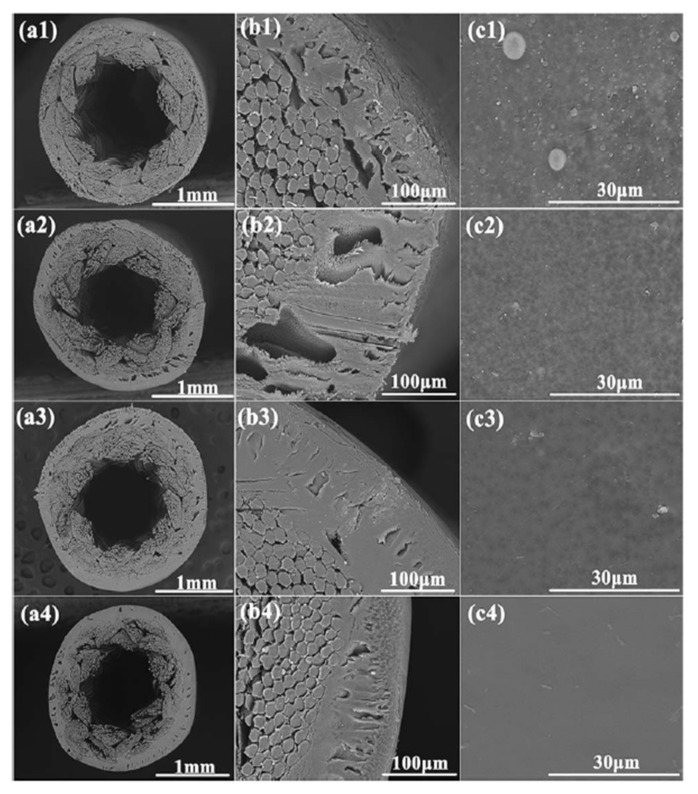
BRHF membrane morphology: (**a**) cross-section;(**b**) cross section partial enlargement; (**c**) outer surface of (**1**) PMIA5 (**2**) PMIA8 (**3**) PMIA10 (**4**) PMIA15 [228]. Reprinted/adapted with permission from Ref. [228]. Copyright 2022, Royal Society of Chemistry.

In a study conducted by Chen et al. [228], the braid reinforced PMIA hollow fiber membrane was fabricated consisting of braid support and a separation layer by using the dry-wet phase inversion technique. In this study, the effects of braid composition and concentration of PMIA on morphology and performance of BR PMIA hollow fiber membrane were analyzed. Figure 23 below shows the morphology of BR PMIA hollow fiber membrane having different concentrations of PMIA in dope solution.

It can be observed that the membrane consists of a braid support and a separation layer. A finger-like porous structure is displayed by the separation layer of BR PMIA hollow fiber membrane in Figure 23b. When the concentration of PMIA is increased, the sponge- like porous structure improves while the finger-like structure disappears. As the concentration of PMIA increases in dope solution, the skin layer becomes dense as shown in Figure 23c. This leads to the conclusion that a high concentration of PMIA in dope solution forms a sponge-like structure and dense skin layer while its low concentration in dope solution forms a finger-like porous structure and a porous skin layer. The viscosity of the dope solution is usually increased with a high concentration of polymer and this result in slowing down the rate of double diffusion between solvent and non-solvent in the process of phase inversion [242]. Membranes having a finger-like porous structure and porous skin layer are to be formed in case of instantaneous demixing whereas sponge-like structures and dense skin layers are formed in case of delayed demixing. During the fabrication process, it is observed that infiltration of dope solution encouraged better interfacial bonding between the separation layer and braided support of membrane. Figure 23c shows the outer surface morphology of the braid reinforced PMIA hollow fiber membrane. It is observed that with an increase in the concentration of PMIA in dope solution, the outer surface became smoother and denser.

**Figure 24:**
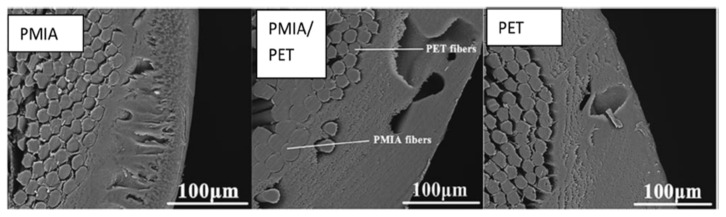
Scanning electron microscopy (SEM) images of PMIA braid, PMIA/PET braid and PET braid [228]. Reprinted/adapted with permission from Ref. [228]. Copyright 2022, Royal Society of Chemistry.

PMIA (poly mphenyleneisophthalamide), PET and PMIA/PET (1:1) braids were used by Chen et al. [228] to produce BRHF membranes by utilizing PMIA polymer Figure 24 [228]. In the case of PMIA fibers, there was a tight bonding between the separation layer of the PMIA braid and the homogenous reinforced braid ensuring good compatibility. On contrary, a weak interfacial bonding in PET reinforced braid is observed as the separation layer and braid were heterogeneous means that there is poor compatibility among PET and PMIA. On using a hybrid braid (PMIA/PET), a separation layer that was created on PET was bonded loosely while the one on PMIA was bonded tightly.

Chen et al., (2017) used BRHF membranes in MBR for the purpose of water filtration [228]. The results indicate that increase in the concentration of PMIA, would result in a decrease in pure water flux and an increase in protein rejection rate. BR PMIA membranes showed excellent interfacial bonding between the reinforcing braid and separation layer as its tensile strength exceeded 170 MPa which indicated its good mechanical property. In the literature, there is hardly any research performed on the use of BRHF membrane in gas separation applications.

## 7. Future Prospects and Concluding Remarks

Previous research work about CO_2_ removal from CH_4_ has highlighted different techniques of separation with their own disadvantages and advantages. Among all methods of gas separation such as absorption, adsorption and cryogenic separation, the membrane-based gas separation process is the most facile, environmentally friendly and simple process. Several spinning parameters were investigated for the fabrication of defect-free membrane. Moreover, according to previous research studies, nanoparticles of ZIFs due to their unique structures show superior adsorption capacity of CO_2_ as compared to the other MOFs.

Some of the advantages of HF membrane include a higher specific surface area along with lower requirements of maintenance and pre-treatment. However, high pressure can cause damage to HF membranes. Braid reinforced HF membranes provide a solution to the problem of mechanical strength faced by hollow fiber membranes. Few research studies are available in the open research literature on this subject. The main focus of these studies is on the utilization of polymer type, braid type, design of spinnerets as well the spinning speed of membrane. This review explains the research conducted on fabricating braid reinforced hollow fiber membranes in previous literature as a summary. One of the most important parameters for fabricating the BRHF membrane is good interfacial bonding between the separation layer and braided support. By selecting polymer types and braids that are compatible with one another, interfacial bonding can be improved. Reinforced HF membranes via employing braids can enhance the mechanical strength of membranes, hence, allowing separations at elevated pressures. Due to their high mechanical strength, they are used in UF membranes in the treatment of wastewater by MBR technology. The literature showed that the trend of the use of BRHF membrane increased in the last ten years. The trend also revealed that the use of reinforced membranes has been varied lately. These days, braid reinforced hollow fiber membranes can be utilized in MBR, UF, RO and NF processes. In the future, research should be performed on the use of braid reinforced hollow fiber membrane in gas separation areas especially for natural gas purification as they have high mechanical strength and it is expected that they can perform well in gas separation areas as they can handle high feed pressures. Moreover, further research should be conducted on the fabrication of braid reinforced HF membranes by employing other techniques such as grafting and blending.

To date, rare studies are reported on the use of BRHF membrane incorporating ZIF-based filler for the purpose of gas separation. Hence, there is a need of conducting further research to analyze the improvement in performance while using BRHF membranes. Furthermore, in the future, the applications of braid reinforced HF membranes are foreseen in various different fields.

## Figures and Tables

**Table 1:** Composition of raw natural gas and pipeline specifications [1,2].

Component	Formula	Composition (mol%)	Maximum Pipeline Specification	Composition
Methane	CH_4_	70–90	Methane	75-none mol%
Ethane	C_2_H_6_	0–20	Ethane	10 mol%
Propane	C_3_ H_8_	0–20	Propane	5 mol%
N-Butane	C_4_ H_10_	2.54	N-Butane	2 mol%
Carbon dioxide	CO_2_	0.1–5	Carbon dioxide	2–3 mol%
Nitrogen	N_2_	0–5	Nitrogen	3 mol%
oxygen	O_2_	0–0.2	Oxygen	0.01 mol%
Hydrogen sulphide	H_2_S	0–5	Hydrogen sulphide	0.25–0.3 g/100 scg
Rare gases	Ar, He, Xe, Ne	trace	Water vapor	4.0–7.0 lb/MM scf

**Table 2:** Commercial membrane materials as well as their selectivities for impurity removal from natural gas [78]. Reprinted/adapted with permission from Ref. [78]. Copyright 2022, Elsevier.

Components Likely to Be Permeated	Preferential Polymeric Material Category	Polymers Utilized	Selectivities over Methane
H_2_S	Rubbery	ether-amide block co-polymer	20–30 (%) *^a^*
CO_2_	Glassy	Polyimide, CA, perfluoropolymer	10–20 (%) *^a^*
N_2_	Rubbery	Silicon rubber	0–3(%) *^a^*
	Glassy	perfluoropolymer	2–3 (%) *^a^*
C_3+_hydrocarbons	Rubbery	Silicon rubber	5–20(%) *^a^*

*^a^* selectivities are typical of those that are measured with high pressure containing natural gas.

**Table 3:** Characteristics, disadvantages and types of polymeric membranes.

Characteristics	(1) Polymer is flexible and soft in a rubbery state while it is hard and rigid in a glassy state. (2) When compared to rubbery membranes, glassy membranes have high glass transition temperature (T_g_) and glassy membranes also have high selectivity CO_2_/CH_4_ [79].
Disadvantages	(1) While handling Carbon dioxide, they might experience plasticization problems. (2) Swelling of the polymer network in the membrane will occur and also segmental mobility increases when the membrane is exposed to CO_2_ which in turn results in an increase in permeability of all the components of gas [80]. (3) Because of this phenomenon, components of gas having characteristics of low permeability will experience high permeability hence the membrane selectivity decreases [70].
Examples	Cellulose acetate, polysulfones, polydimethylsiloxane, polyethersulfone, polyethylene, polyimide, polyether, polypyrrolonesetc

**Table 4:** Advantages and disadvantages of inorganic membranes [84].

Advantages	Disadvantages
Stability in high-pressure applications	Brittleness
Resistance towards high-pressure drop	High operational costs
Easy catalytic activation	Problems in attaining high selectivity in micro porous large-scale membranes.
Resistance towards harsh environmental effects	At high-temperature conditions membrane-to-module sealing becomes difficult.
Easy cleaning	At medium temperature, permeability of highly selective dense membranes is low

**Table 5:** Promising MMMs for purification of natural gas.

Material	*P*_CH_4__	*P*_CO_2__	α_CO_2_/CH_4__	References
Pure Matrimid	0.21	7.29	34.71	[9,158]
Matrimid + MOF-5	0.45	20.20	44.89	[9,138]
Matrimid + CMS	0.24	12.60	52.5	[9]
Pure PSf	0.22	6.30	28.64	[159,160]
PSf + AlPO	1.30	51.00	39.3	[160,161]
Pure ABS	0.12	2.87	24.10	[162]
ABS + AC-2	0.41	20.50	50.10	[163]

**Table 6:** Gas separation performance of MMMs in comparison to pristine polymeric membranes.

Polymer	Filler Used	Filler Loading (wt%)	Gaseous Pair	Pure Polymeric Membrane		Matrix Membranes		References
				Permeability (GPU)	Selectivity	Permeability (GPU)	Selectivity	
Polysulfone	ZIF-8	1	CO_2_/CH_4_	21.4	19.5	31.3	13.5	[163]
Polysulfone	MIL-125(Ti)	20	CO_2_/CH_4_	9.3	22	29.1	29.5	[164]
Matrimid^®^	SAPO-34	20	CO_2_/CH_4_	4.3	34	6.8	67	[165]
6FDA-ODA	UiO-66	7	CO_2_/CH_4_	25.8	20.2	43.3	56.9	[166]
PDMS	4A	50	H_2_/CH_4_	1200	0.8	13,700	14.7	[167]
Pebax 1657	ZIF-8	8	CO_2_/CH_4_	130	9	450	15	[168]
Polyethersulfone	SAPO-34	20	CO_2_/CH_4_	0.9	32.2	2.1	40.5	[169]
Matrimid^®^	ZIF-8	10	H_2_/CH_4_	34	32	25	50	[170]
6FDA-durene	ZIF-8	42	CO_2_/CH_4_	256	19.4	779	20.8	[171]
Pebax 1657	SAPO-34	50	CO_2_/CH_4_	110	18	320	18	[172]

**Table 7:** BRHF membrane production parameters and their effects.

Parameters	Examples	Effect
Polymer type	PS, PVDF, CA, PES, PAN, PAI, PMIA, PI, PSF, PVC	On interfacial bonding between polymer and braid
Support layer	Heterogeneous, Homogeneous and hybrid braid types	On interfacial bonding between polymer and braid
Spinneret design	Diameter of nozzle for coating layer	On membrane morphology and performance, also determines the thickness of coating layer on braid support
Speed of spinning	Fabrication speed	On thickness of coating layer, pore size distribution
Coagulation bath	Temperature	Pore size distribution, morphology and performance

## Data Availability

Not applicable.

## Glossary

ABS	Acrylonitrile Butadiene Styren
AC	Activated Carbons
ALPO	Aluminophosphate
AMDEA	Activated Methyl Diethanolamine
BET	Brunauer–Emmett–Teller
BRHF	Braid Reinforced Hollow Fiber Membranes
CA	Cellulose Acetate
CMS	*Carbon Molecular Sieves*
CNT	Carbon Nanotubes
DEA	Diethanolamine
DEF	N,N-*Diethylformamide*
DMAC	Dimethylacetamide
DMF	Dimethylformamide
FS	Fumed Silica
HF	Hollow Fibers
HMR	Homogeneous *braid*-reinforced
HTR	Heterogeneous braid-*reinforced*
KOH	*Potassium hydroxide*
LBM	Liquefied Biomethane
LNG	Liquefied Natural Gas
MBR	Membrane bioreactor
MDEA	Methyl Diethanolamine
MEA	Monoethanolamine
MF	Microfiltration
MMM	Mixed Matrix Membranes
MOF	Metal Organic Frameworks
NF	Nanofiltration
NMP	N-Methyl-2-Pyrrolidone
PAN	Polyacrylonitrile
PC	Polycarbonate
PDMS	Polydimethylsiloxane
PEBAX	Polyether block amide
PEG	Polyethylene Glycol
PES	Polyethersulfone
PET	Polyethylene Terephthalate
PI	Polyimide
PMIA	Poly(m-phenylene isophthalamide)
PMO	Periodic Mesoporous Organosilica
PSA	Pressure Swing Adsorption
PSF	Polysulfone
PTFE	*Polytetrafluoroethylene*
PTMSP	Poly(1-trimethylsilyl-1-propyne)
PVC	*Polyvinyl Chloride*
PVDF	Polyvinylidene fluoride
PVP	Polyvinylpyrrolidone
PZ	Piperazine
RO	Reverse Osmosis
RTO	Regenerative Thermal Oxidation
SAPO	Silico-Alumino-Phosphate
TFC	Thin Film Composite
TIPS	Thermally Induced Phase Inversion
UF	Ultrafiltration
ZIFs	Zeolite immidazolate frameworks
*Q*	Dope solution feed rate (ml/min)
ρ	dope solution density
ϑ	braid advancing speed
*D_o_*	braid outer diameter
*T*	Dope solution thickness
*A*	pre-exponential factor, i.e., weakly dependent upon temperature
*V**	minimum free volume element size that could accommodate penetrant molecules
γ	overlap factor introduced in order to prevent double counting free volume
*Vf*	average free volume in media that is accessible to transport of penetrants
